# Explainable machine learning for patient‐specific quality assurance in intensity‐modulated radiotherapy based on anatomical structures

**DOI:** 10.1002/acm2.70667

**Published:** 2026-06-24

**Authors:** Xuerou Zhang, Ying Huang, Jie Wang, Xingtong Zhang, Hua Chen, Yehui Luo, Jianhao Xie, Zhiyong Xu, Yunhua Xu

**Affiliations:** ^1^ School of Health Science and Engineering University of Shanghai for Science and Technology Shanghai China; ^2^ Department of Radiation Oncology Shanghai Chest Hospital Shanghai Jiao Tong University School of Medicine Shanghai China; ^3^ Department of Logistics Shanghai Chest Hospital Shanghai Jiao Tong University School of Medicine Shanghai China; ^4^ Yida Technology Service Company Children's Hospital of Fudan University Shanghai China; ^5^ Department of Shanghai Lung Cancer Center Shanghai Chest Hospital Shanghai Jiao Tong University School of Medicine Shanghai China

**Keywords:** gamma passing rate, intensity‐modulated radiation therapy, machine learning, quality assurance, SHAP

## Abstract

**Background:**

Patient‐specific quality assurance (PSQA) plays a pivotal role in intensity‐modulated radiotherapy (IMRT) to ensure accurate dose delivery. However, conventional measurement‐based PSQA approaches are labor‐intensive and provide limited insight into the underlying factors contributing to variations in gamma passing rates (GPRs). Anatomical characteristics of the planning target volume (PTV) and organs at risk (OARs) may contain predictive information relevant to GPR performance, yet their potential has not been fully explored within interpretable machine learning frameworks.

**Purpose:**

This study aimed to develop an interpretable machine learning (ML) framework for predicting GPRs in IMRT based on anatomical features extracted from the PTV and OARs.

**Methods:**

A retrospective cohort of 243 clinical chest IMRT plans was analyzed. Radiomic and dosimetric features were extracted for each anatomical structure. Two ML regression models—Random Forest (RF) and eXtreme Gradient Boosting (XGBoost)—were developed to predict GPRs for the PTV and OARs under four gamma criteria (3%/3 mm, 3%/2 mm, 2%/3 mm, and 2%/2 mm). The GPR obtained by comparing the dose distribution reconstructed using the independent Monte Carlo (MC) dose calculation software ArcherQA (Wisdom Technology Company Limited, Hefei, China)—based on linear accelerator delivery log files—with the original planned dose distribution was used as the reference standard, and calculated using global gamma analysis with a 10% dose threshold. Model performance was evaluated using the mean absolute error (MAE), root mean square error (RMSE), and Spearman's rank correlation coefficient. Shapley Additive Explanations (SHAP) were applied to interpret feature contributions in the best‐performing model.

**Results:**

Both models demonstrated robust predictive performance across different anatomical structures and gamma criteria. As the gamma criteria became less stringent, prediction errors decreased accordingly. Prediction accuracy was relatively high for OARs; for example, under the 3%/3 mm criterion, the test‐set MAE was 0.06% ± 0.01% for the heart and 0.26% ± 0.04% for the whole lung. In contrast, the prediction error was relatively larger for the PTV, with a test‐set MAE of 1.98% ± 0.31% under the same criterion. SHAP analysis revealed that texture‐related radiomic features contributed most substantially to model predictions. Moreover, feature importance patterns varied according to organ type and gamma‐criterion stringency.

**Conclusions:**

Multi‐omics descriptors derived from anatomical structures can reliably predict GPRs in IMRT. The proposed interpretable ML framework not only achieves accurate prediction but also enhances mechanistic understanding through SHAP‐based explanations. These findings provide valuable insights into dose verification variability and offer a practical, transparent tool for IMRT patient‐specific quality assurance.

## 1 INTRODUCTION

The precise delivery of radiation therapy is a primary determinant of patient treatment efficacy and safety.^1^, ^2^ Intensity‐modulated radiation therapy (IMRT) is an advanced radiotherapy technique characterized by both high efficiency and precision. By dynamically adjusting the position of multi‐leaf collimator (MLC) leaves at each fixed irradiation angle, it spatially modulates beam intensity to generate highly conformal dose distributions in three‐dimensional space. This approach maximizes dose coverage within the target area while providing superior protection for surrounding normal organs.^3^, ^4^, ^5^

Nonetheless, the great complexity of this planning is also problematic with respect to dose delivery accuracy. Any small discrepancy in machine parameters, changes in patient anatomy, or any uncertainty in the implementation of the plans may impact the final dose distribution, which may undermine the effectiveness of the treatment and may also result in an increased likelihood of normal tissue complication.^6^, ^7^ Therefore, patient‐specific quality assurance (PSQA) should be carried out on every IMRT plan before being clinically used to ensure that the planned dose matches the delivered dose.^8^, ^9^

The conventional PSQA normally depends on the use of dose measurements, which are done on two or three‐dimensional detector arrays or a phantom. Although these techniques have been broadly used, they tend to be time‐consuming and labor‐intensive in nature.^10^, ^11^, ^12^ As the number of patients attending radiotherapy centers grows and the number of sophisticated treatment plans increases, such a time‐consuming and labor‐intensive process of QA has become a significant bottleneck that hinders the efficiency of the clinical workflow. This has necessitated researchers to find more efficient and automated solutions.

In recent years, PSQA methods based on machine learning (ML) and deep learning (DL) have gained widespread adoption owing to their superior efficiency and speed compared with traditional measurement approaches.^13^ Early studies primarily established gamma passing rate (GPR) prediction models based on treatment plan complexity metrics (e.g., aperture shape), with Valdes et al.^14^ achieving GPR predictions within ± 3% accuracy under the 3%/3 mm gamma criterion using a Poisson Lasso regression model. Subsequently, the research focuses gradually shifted toward feature extraction based on dose distribution. For instance, Park and Hirashima et al.^15^, ^16^ extracted texture features from RT dose to construct GPR prediction models, while Tomori et al.^17^ employed convolutional neural networks (CNNs) to directly learn features from fluence or dose maps.

Ensemble learning has been widely adopted in PSQA for IMRT and VMAT plans due to its ability to effectively integrate multidimensional features, such as treatment complexity and dosimetric characteristics, and its advantages in handling nonlinear relationships, high‐dimensional data, and preventing overfitting.^18^, ^19^ Ensemble learning methods are primarily categorized into bagging and boosting approaches, both of which enhance model performance through distinct mechanisms. Bagging reduces model variance by training multiple models in parallel and aggregating their results, thereby improving the model's generalization capability. Among bagging‐based methods, Random Forest (RF) is frequently employed in QA prediction. Lam et al.^20^ demonstrated that, in dose‐based IMRT QA prediction, the RF achieved 98% of predictions with errors controlled within 3% under the 2%/2 mm gamma criterion. Xue et al.^21^ employed an RF based on plan complexity metrics, achieving a prediction accuracy of up to 98.7%, with a mean absolute error for predicting GPRs as low as 1.23% under the 2%/2 mm gamma criterion. On the other hand, boosting methods systematically reduce bias by training models sequentially, thereby enabling subsequent models to focus on correcting errors made by preceding models. Among these approaches, eXtreme Gradient Boosting (XGBoost) and Adaptive Boosting (AdaBoost) are widely adopted due to their high predictive accuracy and computational efficiency. Hirashima et al.^16^combined plan complexity features with three‐dimensional dosimetric features to construct an XGBoost‐based hybrid prediction model, achieving an average absolute error of 4.2% under the 2%/2 mm standard. Ishizaka et al.^22^ employed a tree‐based ensemble model to handle high‐dimensional radiomic features derived from three‐dimensional dose distributions, yielding model‐predicted root mean square error (RMSE) and mean absolute error (MAE) values ranging from 1% to nearly 10% across the 1%/1 mm, 1%/2 mm, 2%/1 mm, and 2%/2 mm gamma criteria. Thongsawad et al.^23^ employed both AdaBoost and bagged regression trees alongside MLC features, achieving a sensitivity of 94.1% for fault detection in head‐and‐neck radiotherapy plans under the 2%/2 mm criterion. However, despite significant progress in these studies, existing methods still exhibit certain limitations. First, most current models are trained solely on homogeneous phantom data, which makes it difficult to fully capture the complex dose distribution characteristics in real patients. As reported by Ishizaka et al.,^22^ models trained solely on virtual phantoms exhibit significantly reduced prediction accuracy when applied to real patient CT images, primarily owing to the heterogeneity of patient anatomy and dose distribution. Second, existing research predominantly focuses on evaluating overall dose distributions, with relatively few studies addressing GPRs for the PTV and OARs. Furthermore, existing studies have supported three‐dimensional dose verification based on patient anatomy.^24^ Additionally, the inherent black‐box nature of ML/DL has hindered previous QA prediction studies from elucidating the underlying logic of model decision‐making.^25^ This lack of interpretability significantly undermines the credibility and acceptance of models in rigorous clinical settings.

Based on the aforementioned research context and existing challenges, this study proposes an explainable prediction method for GPRs in different anatomical structures during IMRT dose verification, leveraging multi‐omics features. Radiomic and dosimetric features are extracted from patient CT images and RT dose, respectively, to develop an explainable ensemble model for GPR prediction. This study aims to simplify the workflow for clinical physicists and improve the intuitiveness of the treatment planning optimization process.

## 2 MATERIALS AND METHODS

### 2.1 Clinical plans

**FIGURE 1:**
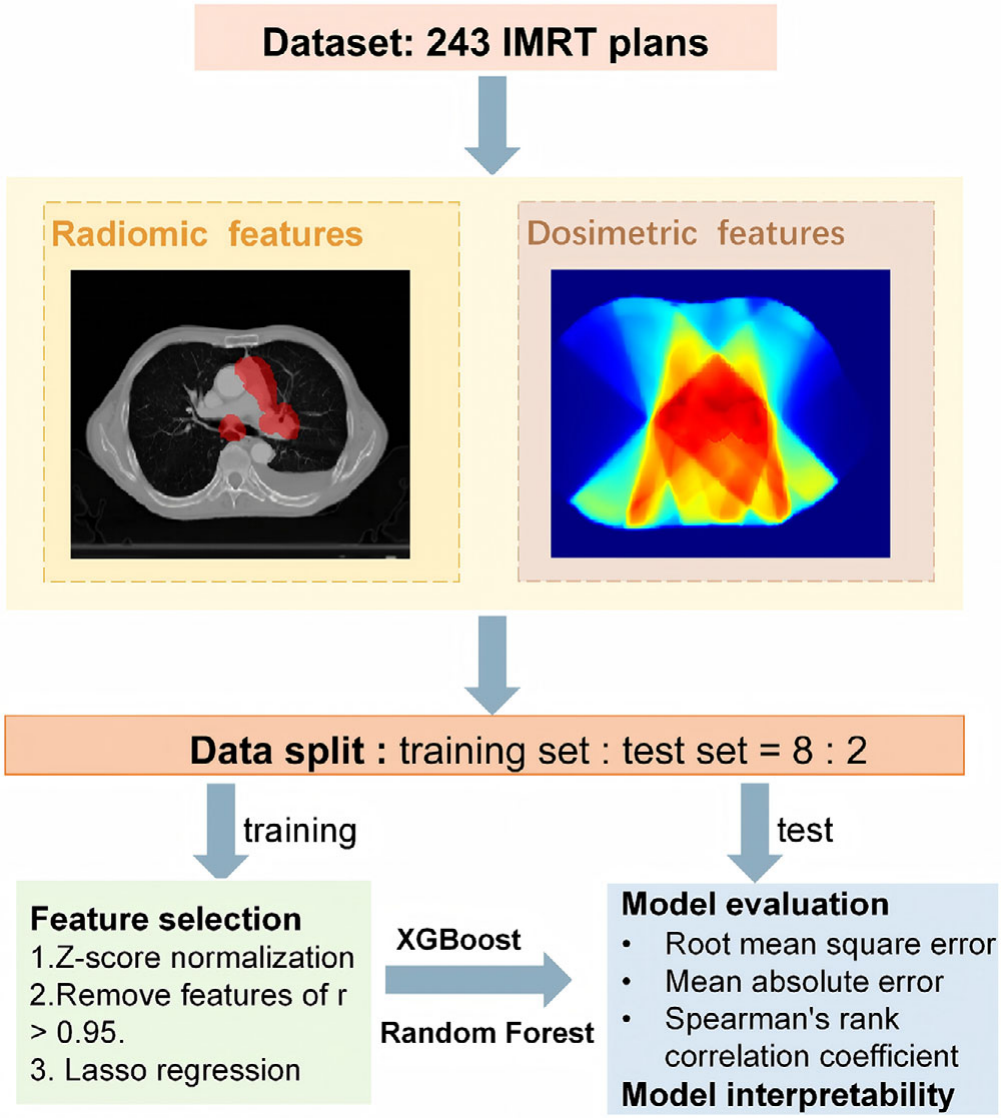
Workflow of the study. The pipeline depicts the process from dataset preparation, through feature engineering, to model training with Random Forest/XGBoost, performance validation, and model interpretability.

The overall workflow of the study is illustrated in Figure 1. This retrospective study included 243 thoracic treatment plans delivered via IMRT at our institution between June 2023 and June 2025. Among these, 100 cases involved lung cancer (60 Gy/30 fractions), and 143 cases involved esophageal cancer (73 cases: 41.4 Gy/23 fractions; 23 cases: 50.4 Gy/28 fractions; 47 cases: 60.2 Gy/28 fractions). All treatment plans were designed using the Pinnacle planning system (Version 16.2, Philips Radiation Oncology Systems, Fitchburg, WI, USA). All plans were delivered using a 6 MV photon beam. The number of beams typically ranged from 3 to 12, with a total of 528.67 ± 142.39 monitor units (MU). Beam arrangements followed standard clinical practice. All plans were generated using default gantry rotation speeds and MLC motion parameters. All treatment plans were developed according to standardized clinical workflows and are representative of typical plan complexity under routine clinical conditions. Dose calculations employed the direct machine parameter optimization (DMPO) algorithm with a resolution of 1.0 mm. Treatments were delivered using a Varian Edge linear accelerator equipped with a high‐definition Millennium 120 MLC collimator comprising two rows of 60 leaves, with the outer 28 leaves and inner 32 leaves measuring 0.5 cm and 0.25 cm in width, respectively. All planning CTs used in this study were acquired under free‐breathing conditions, which is the standard clinical practice for thoracic IMRT simulation at our institution. All CT simulation images were acquired using a Siemens Somatom Definition AS multi‐slice spiral CT scanner. The scanning parameters were as follows: tube voltage 120 kV, tube current 140 mA, scan time 14.52 s, rotation time 1 s/rot, pitch 1.2, and convolution kernel B30s (medium smooth). All IMRT plans were delivered on this system, and log files were recorded during delivery. This study was approved by the Institutional Review Board of our institution.

### 2.2 Radiomics and dosimetric feature extraction

Radiomic and dosimetric features were extracted from CT images and the corresponding RT dose, respectively. Regions of interest (ROIs) included the heart, left lung, right lung, total lung, spinal cord, and PTV. PTV was outlined by doctors according to institutional protocols. For critical organs such as the lungs, heart, and spinal cord, the initial contours were generated using the built‐in automatic contouring script of the Pinnacle treatment planning system. Afterwards, experienced radiation oncologists visually inspected and manually adjusted these automatically generated contours to ensure anatomical accuracy. The ROIs used in this study were derived directly from the clinical contours provided in the RT dose data, without any morphological operations. During feature extraction, the PTV was treated as the priority region. In cases of overlap between the PTV and OARs, features were calculated based on the PTV. Feature extraction was performed using the open‐source Python library Pyradiomics (v3.1.0).^26^

To enhance the feature extraction process and optimize contrast display for different organs, window width and level were pre‐set based on the CT value ranges of each organ. A total of 107 radiomics features were extracted from the preprocessed CT scans, and 107 dosimetric features were extracted from the RT dose files. All Pyradiomics parameters and dose feature extraction settings are listed in Table S‐1. All extracted features were categorized into three groups: (I) Shape features (14 features): describe the three‐dimensional geometric properties of regions of interest. (II) First‐order features (18 features): first‐order statistics based on voxel intensity values, reflecting the intensity distribution within a region. (III) Texture features (75 features): extracted via the gray‐level dependence matrix (GLDM, 14 features), gray‐level co‐occurrence matrix (GLCM, 24 features), gray‐level run length matrix (GLRLM, 16 features), gray‐level size‐zone matrix (GLSZM, 16 features), and neighboring gray‐tone difference matrix (NGTDM, 5 features) to quantify higher‐order spatial patterns of intensity. These features collectively provide comprehensive information about the ROIs in terms of shape, intensity statistics, and spatial texture. Ultimately, all features were consolidated into a CSV file to serve as input data for subsequent ML model training.

### 2.3 Dose verification

Independent dose calculations were performed using the commercial MC dose engine ArcherQA (Wisdom Technology Company Limited, Hefei, China, Version 1.0).^27^ ArcherQA reconstructs the delivered dose distribution using an MC algorithm based on linear accelerator delivery log files and compares it with the planned dose distribution to evaluate dose agreement. The ArcherQA dose verification system has undergone rigorous local commissioning following the acceptance testing and commissioning guidelines outlined in the American Association of Physicists in Medicine (AAPM) Task Group 119 (TG‐119), TG‐157, and TG‐219 reports,^7^, ^28^, ^29^ as well as relevant literature.^30^, ^31^ Specifically, percentage depth dose (PDD) and off‐center ratio (OCR) data were calculated using the ArcherQA dose verification system model and compared with water tank measurement data to verify the accuracy of the beam model. All 243 verification plans (RT plans, RT structures, RT doses, and CT images) were imported into ArcherQA, where MC algorithms recalculated dose distributions. Under various gamma criteria (3%/3 mm, 3%/2 mm, 2%/3 mm, 2%/2 mm), GPRs were predicted by comparing MC‐calculated dose distributions with planned dose distributions.

During gamma analysis, a global gamma method was applied, with dose comparison based on absolute dose values without any normalization. Linear interpolation was used for spatial interpolation. The TPS used a 2.5 mm grid for dose calculation and comparison. Region‐of‐interest masking was performed on a per‐structure basis, with gamma analysis conducted separately for the PTV and each OAR. A low‐dose threshold of 10% was applied, also on a per‐structure basis.

### 2.4 Construction and evaluation of the gamma pass rate prediction model

The selected features were incorporated into two representative ensemble machine learning methods—RF (v1.0.2) and XGBoost (v2.1.4)—to construct prediction models. Under the same gamma criterion, we trained and evaluated the PTV and OAR parallel models separately, and calculated the RMSE using a weighted average method to optimize the overall performance. To evaluate model performance, the dataset was randomly divided into training and test sets at an 8:2 ratio. This random partitioning was repeated 100 times, and the average results were calculated to ensure a representative data distribution. Model training and feature selection were performed exclusively on the training set, with RMSE used as the scoring metric to select the best model, while the test set was kept strictly independent and used only for final performance evaluation. To reduce feature dimensionality, minimize redundant information, and improve the model's generalization capability, a feature selection procedure was implemented on the training set. First, features in the training set were standardized using Z‐scores. Subsequently, a two‐stage feature screening strategy was adopted. In the first stage, correlation coefficients between all feature pairs in the training set were calculated. For feature pairs with an absolute correlation coefficient greater than 0.95, one feature was removed to reduce multicollinearity. In the second stage, the Least Absolute Shrinkage and Selection Operator (LASSO) regression model was applied, with the penalty parameter optimized through cross‐validation to select the subset of features with non‐zero coefficients. LASSO regression analysis was performed using the Python scikit‐learn library (version 1.0.2).

To comprehensively evaluate the model's predictive performance and robustness on unseen data, multiple regression metrics were employed for quantitative analysis, including MAE, RMSE, and Spearman's rank correlation coefficient.

### 2.5 Model interpretation and visualization

To gain deeper insights into the predictive mechanisms of the optimal ensemble model, this study employed the SHAP method for quantitative interpretation. SHAP analysis, grounded in game theory principles, consistently assigns each feature's contribution to model outputs.^32^, ^33^ Specifically, SHAP dependence plots were generated for key features related to the PTV and OARs to illustrate the relationship between changes in feature values and predicted GPR, as well as potential feature interaction effects. In addition, feature stability was assessed across 100 repeated resampling runs. A higher selection frequency indicated greater stability across these runs, reflecting higher robustness and potential importance for model prediction. A higher selection frequency indicated greater stability across these runs, reflecting higher robustness and potential importance for model prediction. Through SHAP analysis and feature selection frequency statistics, this study interpreted the basis of model prediction from both single‐feature contribution and overall stability perspectives.

## 3 RESULTS

### 3.1 GPR results based on dose reconstructed from ArcherQA MC log files

Table 1 presents the range, mean, median, and standard deviation (SD) of the GPRs for the PTV, left lung, right lung, total lung, heart, and spinal cord, obtained by comparing the dose reconstructed from ArcherQA MC log files with the planned dose under the criteria of 3%/3 mm, 3%/2 mm, 2%/3 mm, and 2%/2 mm. As the gamma criteria became more stringent, indicated by smaller dose differences and distance‐to‐agreement values, the overall mean and median GPRs generally decreased, whereas the range and standard deviation increased. PTV GPR values were generally lower than those of other organs, whereas the lungs, heart, and spinal cord exhibited higher GPR values. SD reflects the dispersion of GPR values. For the PTV, SD increased as the standards became stricter (from 3.48% to 7.50%), whereas SD for other organs showed minimal variation.

**TABLE 1:** Mean, median, range, and SD gamma passing rates of the planning target volume and organs at risk based on dose reconstructed from ArcherQA MC log files.

Organ		Mean [%]	Median [%]	Range [%]	SD [%]
PTV	3%/3 mm	96.2	97.2	75.4–99.9	3.48
	3%/2 mm	92.3	93.4	67.5–99.0	4.79
	2%/3 mm	91.4	92.7	57.4–98.6	5.82
	2%/2 mm	83.8	85.1	46.7–95.8	7.50
Lung_L	3%/3 mm	99.6	99.9	90.7–100.0	0.99
	3%/2 mm	99.1	99.9	85.8–100.0	1.86
	2%/3 mm	99.0	99.8	85.1–100.0	1.79
	2%/2 mm	98.0	99.5	79.1–100.0	3.20
Lung_R	3%/3 mm	99.7	99.9	94.5–100.0	0.64
	3%/2 mm	99.2	99.8	89.1–100.0	1.34
	2%/3 mm	99.2	99.8	91.4–100.0	1.25
	2%/2 mm	98.1	99.2	84.1–100.0	2.51
Total Lung	3%/3 mm	99.7	99.8	96.4–100.0	0.47
	3%/2 mm	99.2	99.5	94.4–100.0	0.91
	2%/3 mm	99.2	99.5	94.5–100.0	0.84
	2%/2 mm	98.2	98.7	91.9–100.0	1.52
Heart	3%/3 mm	99.9	99.9	99.2–100.0	0.09
	3%/2 mm	99.8	99.9	98.8–100.0	0.22
	2%/3 mm	99.8	99.9	98.6–100.0	0.26
	2%/2 mm	99.4	99.7	86.8–100.0	1.04
Spinal cord	3%/3 mm	99.9	100.0	99.9–100.0	0.13
	3%/2 mm	99.8	99.9	97.3–100.0	0.38
	2%/3 mm	99.7	99.9	97.6–100.0	0.43
	2%/2 mm	99.1	99.4	92.7–100.0	1.05

Abbreviations: Lung_L: left Lung; Lung_R: right Lung; PTV: planning target volume; SD: standard deviation.

### 3.2 Performance evaluation of machine learning models

#### 3.2.1 Regression prediction performance under different gamma standards

Table 2 and Table 3 present the RMSE, MAE, and Spearman's rank correlation coefficient for the predicted GPRs under the 3%/3 mm, 3%/2 mm, 2%/3 mm, and 2%/2 mm criteria, based on the RF and XGBoost models. Overall, all models demonstrated significantly higher predictive accuracy for OARs than for the PTV. When comparing the two algorithms, the XGBoost model demonstrated relatively superior performance, exhibiting lower prediction errors. Under the 3%/3 mm standard, prediction errors varied substantially across anatomical structures. The PTV showed the largest prediction error, with the lowest MAE of 1.98% ± 0.31%, whereas the prediction errors for OARs were markedly lower, particularly for the heart and spinal cord, with MAE values below 0.06% ± 0.01%. As the gamma criteria became stricter, the prediction errors of both models increased significantly. With respect to the rank correlation between predicted and reference values, an overall downward trend was observed. The left lung exhibited the highest correlation under the 2%/2 mm criterion, with a Spearman correlation coefficient of 0.75. Conversely, the spinal cord exhibited the lowest Spearman correlation coefficient across multiple criteria, indicating the lowest predictive stability.

**TABLE 2:** Regression metrics for planning target volume predictions under different gamma criteria (test set).

Organ		MAE (RF) (%)	RMSE (RF) (%)	Sr (RF)	MAE (XGBoost) (%)	RMSE (XGBoost) (%)	Sr (XGBoost)
PTV	3%/3 mm	1.98 ± 0.31	2.95 ± 0.56	0.52	1.99 ± 0.34	3.04 ± 0.66	0.52
	3%/2 mm	2.83 ± 0.40	4.00 ± 0.67	0.59	2.90 ± 0.46	4.14 ± 0.80	0.58
	2%/3 mm	3.62 ± 0.51	4.95 ± 0.82	0.46	3.60 ± 0.52	4.98 ± 0.90	0.45
	2%/2 mm	4.97 ± 0.62	6.59 ± 0.84	0.48	5.06 ± 0.68	6.79 ± 1.05	0.47

Abbreviations: MAE: mean absolute error; MSE: mean squared error; PTV: planning target volume; RF: Random Forest; RMSE: root mean squared error; Sr: Spearman rank correlation coefficients; XGBoost: eXtreme Gradient Boosting.

**TABLE 3:** Regression metrics for organs at risk predictions under different gamma criteria (test set).

Organ		MAE (RF) (%)	RMSE (RF) (%)	Sr (RF)	MAE (XGBoost) (%)	RMSE (XGBoost) (%)	Sr (XGBoost)
Lung_L	3%/3 mm	0.38 ± 0.10	0.82 ± 0.28	0.72	0.35 ± 0.09	0.76 ± 0.29	0.73
	3%/2 mm	0.71 ± 0.16	1.37 ± 0.37	0.71	0.66 ± 0.16	1.28 ± 0.39	0.73
	2%/3 mm	0.72 ± 0.16	1.37 ± 0.39	0.74	0.66 ± 0.15	1.25 ± 0.39	0.75
	2%/2 mm	1.28 ± 0.23	2.21 ± 0.47	0.75	1.22 ± 0.24	2.10 ± 0.49	0.75
Lung_R	3%/3 mm	0.29 ± 0.06	0.53 ± 0.16	0.59	0.29 ± 0.07	0.58 ± 0.17	0.59
	3%/2 mm	0.61 ± 0.12	1.08 ± 0.29	0.63	0.61 ± 0.12	1.09 ± 0.31	0.62
	2%/3 mm	0.55 ± 0.11	0.92 ± 0.21	0.65	0.55 ± 0.11	0.92 ± 0.21	0.65
	2%/2 mm	1.13 ± 0.20	1.76 ± 0.35	0.67	1.11 ± 0.19	1.73 ± 0.32	0.69
Total lung	3%/3 mm	0.26 ± 0.04	0.44 ± 0.10	0.63	0.26 ± 0.05	0.44 ± 0.11	0.63
	3%/2 mm	0.52 ± 0.08	0.81 ± 0.15	0.65	0.53 ± 0.08	0.85 ± 0.16	0.64
	2%/3 mm	0.46 ± 0.07	0.70 ± 0.13	0.68	0.47 ± 0.07	0.73 ± 0.14	0.68
	2%/2 mm	0.85 ± 0.11	1.19 ± 0.18	0.70	0.87 ± 0.11	1.22 ± 0.18	0.68
Heart	3%/3 mm	0.06 ± 0.01	0.09 ± 0.02	0.27	0.06 ± 0.01	0.09 ± 0.03	0.29
	3%/2 mm	0.16 ± 0.02	0.23 ± 0.03	0.28	0.16 ± 0.02	0.23 ± 0.03	0.26
	2%/3 mm	0.19 ± 0.03	0.28 ± 0.04	0.41	0.18 ± 0.02	0.25 ± 0.04	0.45
	2%/2 mm	0.50 ± 0.10	0.93 ± 0.47	0.55	0.50 ± 0.10	0.98 ± 0.47	0.53
Spinal cord	3%/3 mm	0.09 ± 0.01	0.15 ± 0.03	0.03	0.08 ± 0.01	0.13 ± 0.03	0.04
	3%/2 mm	0.26 ± 0.04	0.40 ± 0.07	0.07	0.25 ± 0.04	0.41 ± 0.10	0.01
	2%/3 mm	0.29 ± 0.04	0.44 ± 0.09	0.09	0.29 ± 0.04	0.47 ± 0.09	0.04
	2%/2 mm	0.71 ± 0.10	1.02 ± 0.19	0.25	0.69 ± 0.10	1.01 ± 0.20	0.22

Abbreviations: Lung_L: left Lung; Lung_R: right Lung; MAE: mean absolute error; MSE: mean squared error; RF: Random Forest; RMSE: root mean squared error; Sr: Spearman rank correlation coefficients; XGBoost: eXtreme Gradient Boosting.

#### 3.2.2 Scatter distribution of predicted and reference values

**FIGURE 2:**
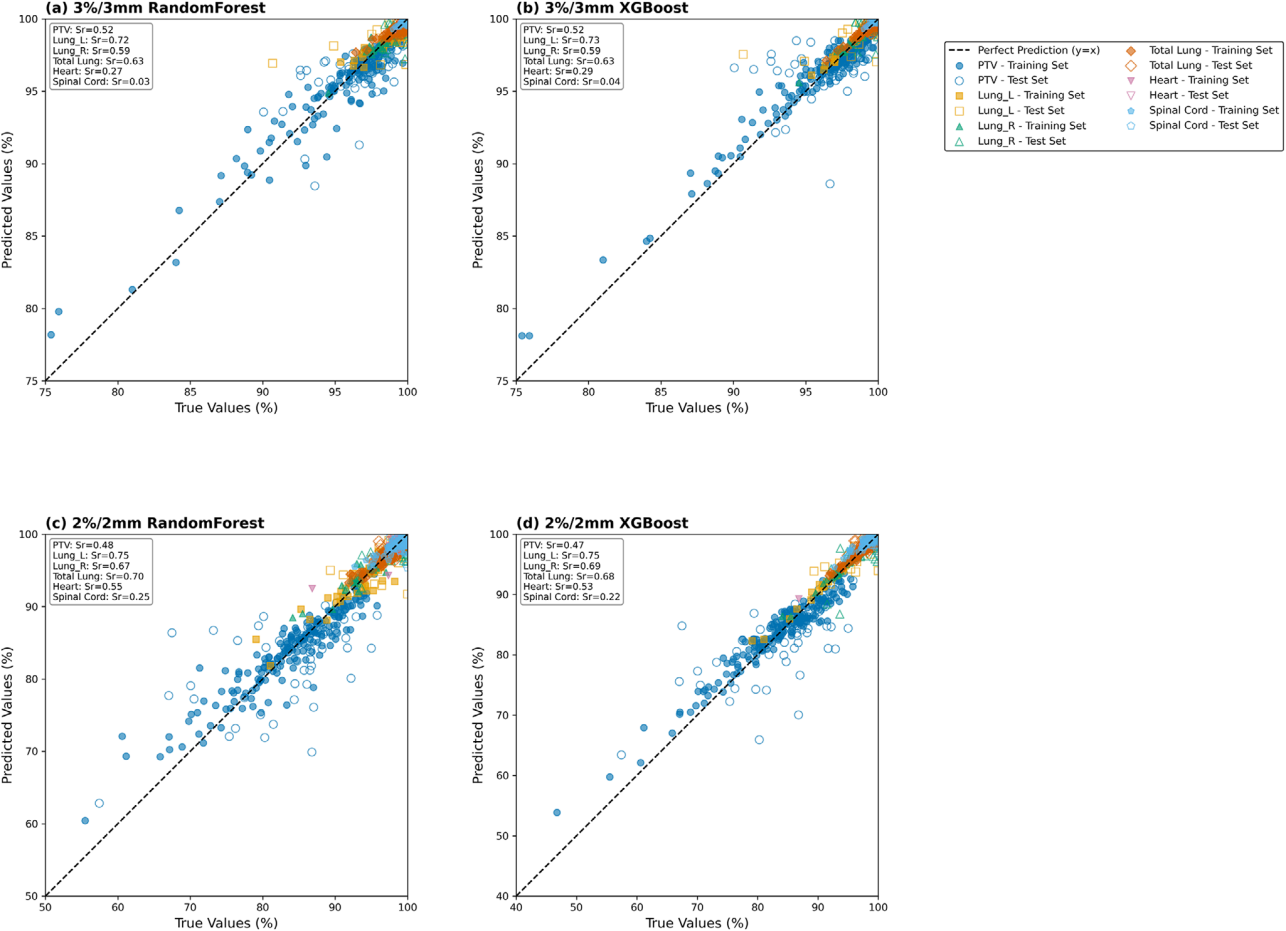
Scatter distribution plots of the Random Forest and XGBoost models under the 3%/3 mm and 2%/2 mm gamma criteria: (a) 3%/3 mm Random Forest; (b) 3%/3 mm XGBoost; (c) 2%/2 mm Random Forest; (d) 2%/2 mm XGBoost.

Figure 2 shows the scatter distribution of predicted GPRs versus reference GPRs for the RF and XGBoost models under the 3%/3 mm and 2%/2 mm criteria. Results for the remaining criteria (3%/2 mm and 2%/3 mm) are provided in the Figure S‐1.

Under all gamma criteria within the PTV, the predicted GPR values exhibited a clear linear correlation with the reference GPRs. Predictions for high GPR values clustered closer to the ideal diagonal line, whereas points for low GPR values showed a noticeable leftward shift, indicating a systematic tendency of the model to overestimate PTV values at the lower end of the GPR spectrum.

Among all organs, the total lung exhibited the most ideal predictive performance, with predicted values closely consistent with reference GPRs. Data points clustered tightly around the ideal fit line, exhibiting minimal dispersion and no discernible systematic bias, resulting in substantially higher predictive accuracy compared with the PTV. The left and right lungs also demonstrated good fitting performance. In contrast, although the predicted values for the heart maintained a positive correlation with reference GPRs, the dispersion of data points was markedly greater than that observed for the lungs. Particularly in regions with lower GPR values, some predicted values deviated from the diagonal line, indicating a certain degree of prediction error in these regions. The predicted values for the spinal cord exhibited a narrower distribution range and generally followed the trend of reference GPRs. However, under strict gamma criteria, some data points deviated from the expected trend, resulting in lower predictive accuracy compared with the lungs.

#### 3.2.3 Predictive distribution characteristics and consistency analysis

To clearly present the boundary performance of the model, this study focuses on the results under the 3%/3 mm and 2%/2 mm gamma criteria; the corresponding figures for the remaining criteria, namely 3%/2 mm and 2%/3 mm, are provided in the Figures S‐2–S‐6. Since the XGBoost model generally outperformed the Random Forest model in terms of regression evaluation metrics, only the results of the XGBoost model are presented in this subsection.

**FIGURE 3:**
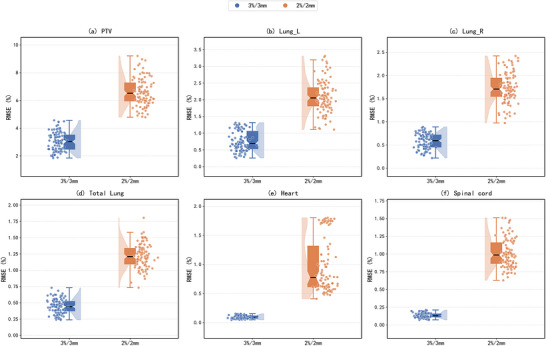
Distribution plots of prediction errors for the PTV and organs at risk using the XGBoost model under the 3%/3 mm and 2%/2 mm gamma criteria: (a) PTV; (b) left lung; (c) right lung; (d) total lung; (e) heart; (f) spinal cord.

Figure 3 shows the distribution of prediction errors, expressed as RMSE, for the PTV and each OAR using the XGBoost model under the 3%/3 mm and 2%/2 mm gamma criteria. Overall, when the gamma criterion changed from 3%/3 mm to 2%/2 mm, the error levels for both the PTV and OARs increased markedly, and the overall distribution range became wider, indicating increased prediction difficulty and greater error dispersion under stricter gamma criteria. For different anatomical structures, the PTV showed the highest overall error, with a wider distribution under the 2%/2 mm criterion, suggesting that prediction of PTV GPR was more strongly affected by stricter criteria. The error distributions for the left lung, right lung, and total lung were relatively concentrated. Among them, the total lung showed good stability under both criteria, although the median and upper range of errors still increased under the stricter criterion. In contrast, the heart and spinal cord showed relatively low errors under the 3%/3 mm criterion, but their dispersion increased noticeably under the 2%/2 mm criterion, suggesting reduced prediction stability for these structures under stricter conditions.

**FIGURE 4:**
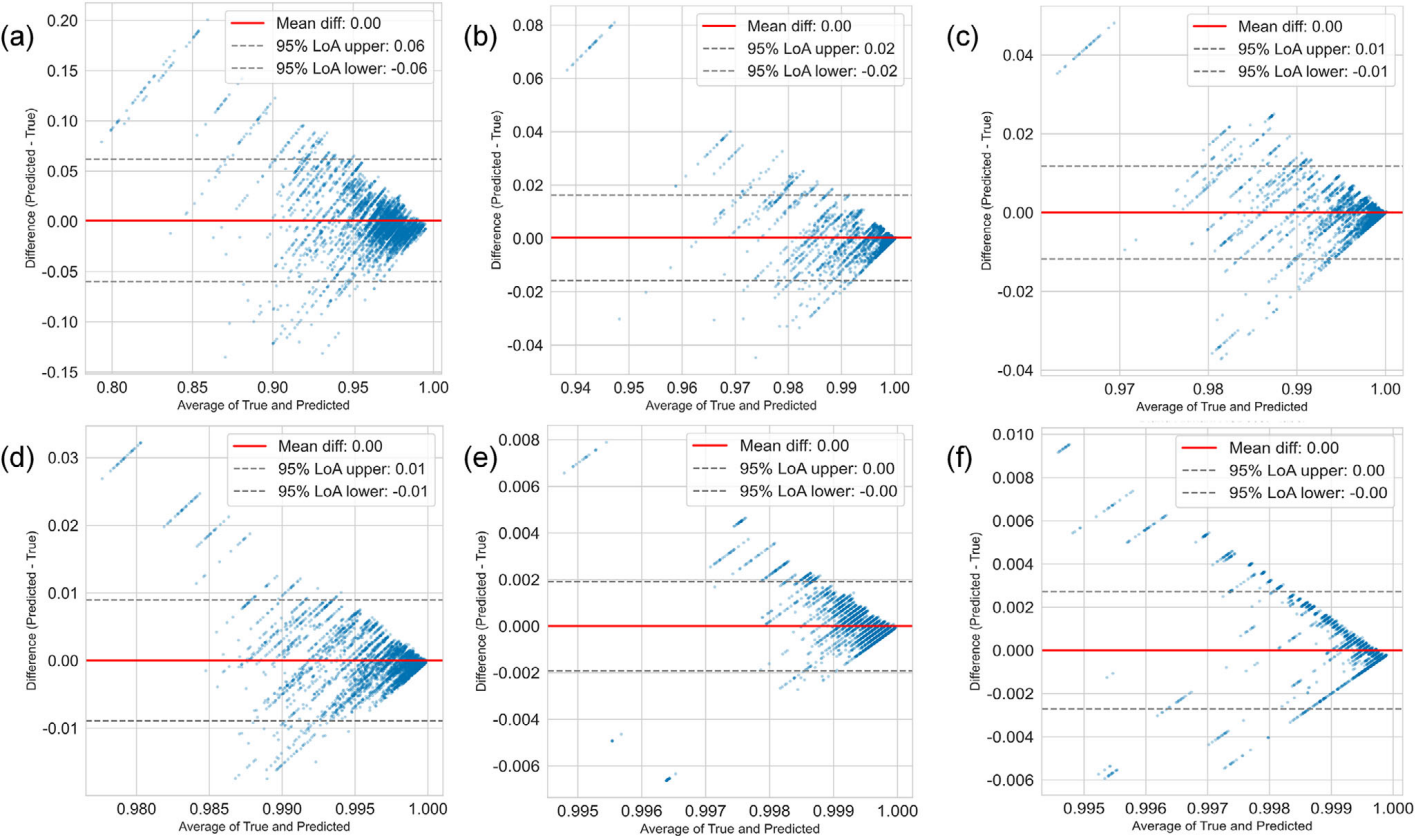
Bland–Altman analysis of XGBoost under the 3%/3 mm gamma criterion: (a) PTV; (b) left lung; (c) right lung; (d) total lung; (e) heart; (f) spinal cord.

**FIGURE 5:**
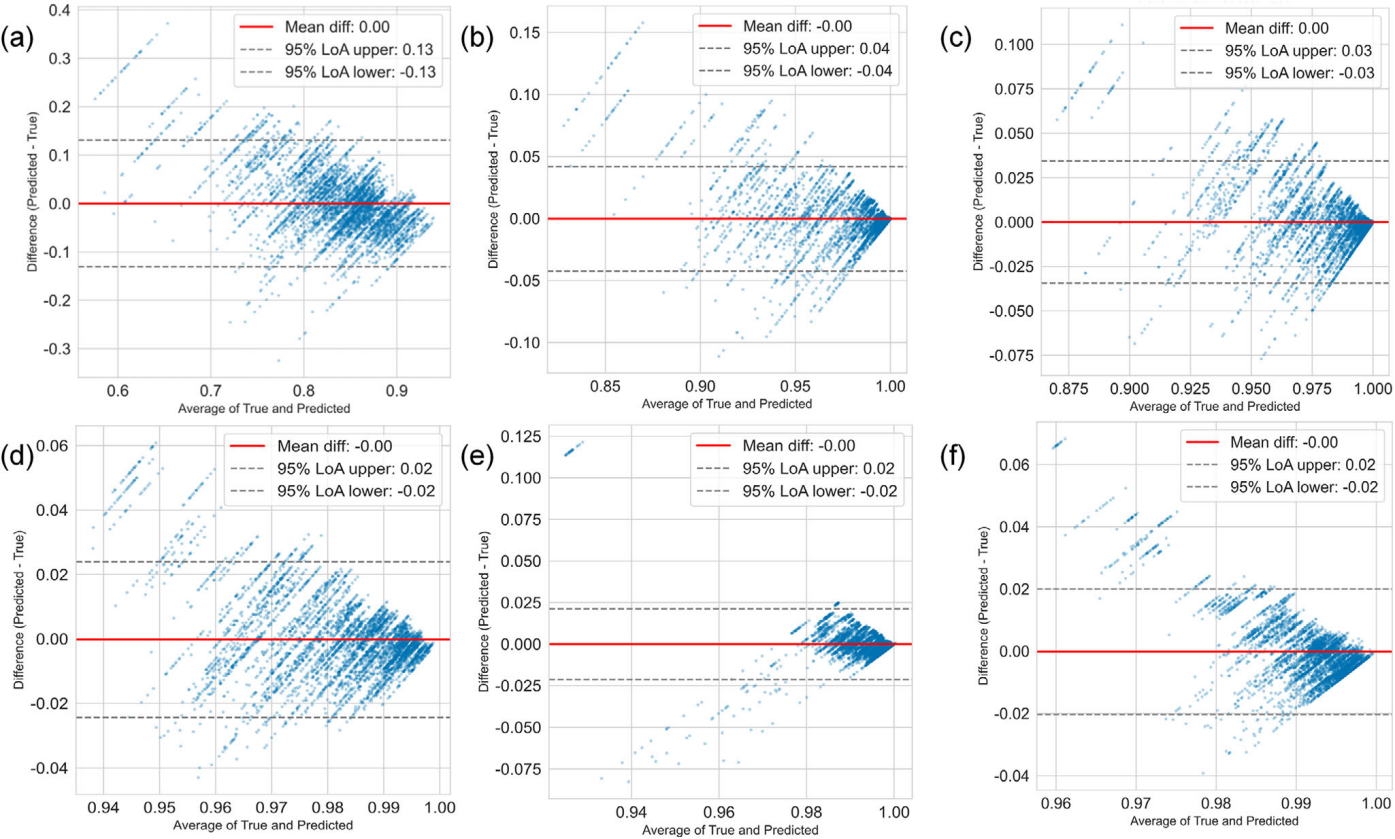
Bland–Altman analysis of XGBoost under the 2%/2 mm gamma criterion: (a) PTV; (b) left lung; (c) right lung; (d) total lung; (e) heart; (f) spinal cord.

Figures 4 and 5 present the Bland–Altman analysis, showing that no significant systematic bias was observed across all anatomical structures, with the mean differences consistently close to 0. The PTV exhibited the widest limits of agreement (LoA), which gradually increased from approximately ± 0.06 to ± 0.13 as the gamma criteria became stricter. This continuous widening suggests increased dispersion of prediction errors and reduced stability under stricter criteria. In contrast, the limits of agreement for the organs at risk were generally narrower, indicating better prediction agreement than that observed for the PTV. Among them, lung‐related metrics maintained good robustness, with LoA values generally distributed within approximately ± 0.01 to ± 0.04. The heart and spinal cord showed the smallest LoA, approximately ranging from ± 0.002 to ± 0.02, suggesting relatively small prediction errors; however, their GPR values were highly concentrated near 100%, resulting in a limited dynamic range.

#### 3.2.4 Comparison of model performance under different feature combinations

To evaluate the impact of different feature combinations on model prediction performance, this study compared three schemes: radiomics features alone, dosiomics features alone, and their combination. Table 4 presents test set RMSE results of the RF and XGBoost models under four gamma criteria. Overall, across all evaluation criteria and models, multi‐omics features achieved the lowest RMSE, outperforming single‐feature inputs. Taking the XGBoost model as an example, under the 3%/3 mm criterion, the test set RMSE of the multi‐omics model was 1.31%, compared with 1.37% for radiomics features alone and 1.73% for dosiomics features alone. Under the stricter 2%/2 mm criterion, the RMSE of the multi‐omics model was 3.08%, again outperforming radiomics features alone at 3.38% and dosiomics features alone at 3.27%. The RF model showed a consistent trend across all criteria.

**TABLE 4:** RMSE values of Random Forest and XGBoost models on the test set under different feature combinations.

Feature		RMSE (RF) (%)	RMSE (XGBoost) (%)
Radiomics features	3%/3 mm	1.33	1.37
	3%/2 mm	1.95	1.96
	2%/3 mm	2.32	2.35
	2%/2 mm	3.35	3.38
Dosiomics features	3%/3 mm	1.69	1.73
	3%/2 mm	2.28	2.32
	2%/3 mm	2.56	2.61
	2%/2 mm	3.26	3.27
Multi‐omics features	3%/3 mm	1.28	1.31
	3%/2 mm	1.82	1.87
	2%/3 mm	2.16	2.16
	2%/2 mm	3.02	3.08

Abbreviations: RF: Random Forest; RMSE: root mean squared error; XGBoost: eXtreme Gradient Boosting.

### 3.3 Model interpretability

**FIGURE 6:**
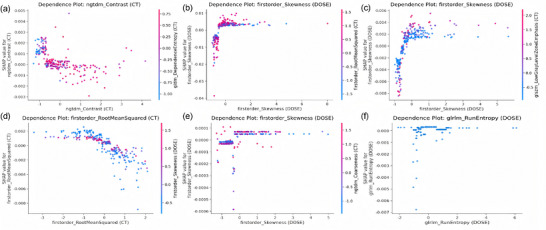
SHAP dependence plots of the main features for XGBoost under the 3%/3 mm gamma criterion: (a) PTV; (b) left lung; (c) right lung; (d) total lung; (e) heart; (f) spinal cord.

Given that 3%/3 mm is a commonly used clinical gamma criterion and that the XGBoost model demonstrated superior overall performance, the main text focuses on representative SHAP feature dependence plots for the PTV and each OAR under this condition, illustrating the effects of key features on the model predictions (Figure 6). The feature selection frequencies for each structure and the SHAP analysis results for the remaining features are provided in the Figures S‐7–S‐13.

Figure 6 shows representative SHAP dependence plots for the PTV, left lung, right lung, total lung, heart, and spinal cord, respectively. In combination with the feature selection results (Figure S‐10), for the PTV, ngtdm_Contrast (CT), gldm_DependenceVariance (CT), and lszm_SizeZoneNonUniformityNormalized (CT) were frequently selected features, suggesting that CT texture heterogeneity features play an important role in model construction. The SHAP dependence plots further showed that, as ngtdm_Contrast (CT) increased, its contribution to the model output generally decreased, indicating that greater local gray‐level differences may be associated with unfavorable dosimetric outcomes. An increase in gldm_DependenceVariance (CT) suggests greater complexity of the texture structure, which may affect the uniformity of dose distribution. In addition, a potential interaction may exist between ngtdm_Contrast (CT) and gldm_DependenceEntropy (CT). In regions with higher contrast, samples with higher entropy generally exhibited lower SHAP values, indicating that when multiple texture complexity features are combined, the model tends to predict a lower GPR.

For the left lung, right lung, and total lung, the feature patterns of the three structures showed a high degree of overall consistency. The frequently selected features mainly included the dose first‐order statistical feature firstorder_Skewness (DOSE), the CT first‐order statistical feature firstorder_RootMeanSquared (CT), and several CT/dose texture features, such as ngtdm_Contrast (DOSE), glszm_ZoneVariance (DOSE), and glszm_SmallAreaLowGrayLevelEmphasis (CT). Among these features, firstorder_Skewness (DOSE) reflects the asymmetry of the dose distribution. The SHAP dependence plots showed that, as this feature increased, its contribution to the model output gradually shifted from negative to positive and tended to stabilize in the higher‐value range, suggesting an association between the asymmetry of the dose distribution and the predicted GPR. firstorder_RootMeanSquared (CT) reflects the overall gray‐level intensity of the lung. As this feature increased, its SHAP values generally decreased, indicating that higher overall gray‐level intensity was associated with a lower predicted GPR. An interaction was also observed between these two features. Specifically, the contribution of dose distribution skewness to the model output varied according to the overall gray‐level intensity of the lung, while the effect of CT gray‐level intensity features on the predicted GPR may also be modulated by dose distribution skewness.

For the heart, the feature selection results showed that firstorder_Skewness (DOSE) was a stably selected feature, suggesting that the asymmetry of the dose distribution in the heart region was associated with the predicted GPR. As firstorder_Skewness (DOSE) increased, its contribution to the model output gradually shifted from negative to positive and then tended to stabilize, indicating that morphological characteristics of the dose distribution influenced the model's assessment of cardiac GPR. In addition, CT texture features such as ngtdm_Busyness (CT), ngtdm_Coarseness (CT), glrlm_RunEntropy (CT), and glszm_SizeZoneNonUniformityNormalized (CT) also showed relatively high selection frequencies, indicating that the internal structural complexity and spatial heterogeneity of the heart made important contributions to the model. A potential interaction may exist between firstorder_Skewness (DOSE) and ngtdm_Coarseness (CT), suggesting that the contribution of dose distribution skewness to the model output may vary with the CT texture coarseness of the heart.

For the spinal cord, glrlm_RunEntropy (DOSE) was the main feature, suggesting that the spatial complexity of the dose distribution in the spinal cord region was closely associated with the predicted GPR. glrlm_RunEntropy (DOSE) exerted a nonlinear effect on the model output and showed a relatively pronounced negative contribution within certain value ranges, indicating that more complex and heterogeneous dose distribution patterns in the spinal cord may be associated with a lower GPR.

Overall, the key radiomic and dosiomics features used by the model were closely associated with the anatomical characteristics of each organ. Across all organs and gamma criteria, features related to texture heterogeneity showed high importance. As the gamma criteria became more stringent, shifting from 3%/3 mm to 2%/2 mm, the distribution of feature importance exhibited a systematic trend: the model relied increasingly on local complex texture features. Under the 3%/3 mm criterion, the model incorporated both relatively simple first‐order statistical features, such as firstorder_Skewness (DOSE), and moderately complex texture features, such as ngtdm_Contrast (CT). Specifically, firstorder_Skewness reflects the overall asymmetry of the dose distribution and represents a global feature, suggesting that under a relatively loose criterion, the overall morphology of the dose distribution is already associated with variations in GPR. In contrast, ngtdm_Contrast characterizes local gray‐level differences and reflects moderate texture complexity. Under the 2%/2 mm criterion, the importance of simple features decreased markedly, while higher‐order complex texture features became the main predictive factors, such as gldm_DependenceVariance (CT). This feature quantifies the variation in gray‐level dependence among neighboring voxels in CT images. A higher value indicates a more irregular texture structure and greater internal heterogeneity. This suggests that under stricter criteria, the model relies more heavily on such complex features to sensitively identify local non‐uniformity and subtle changes in image or dose distributions. Other similarly important complex features include glcm_Contrast and glrlm_RunEntropy.

## 4 DISCUSSION

This study developed an ML model based on multi‐omics features to predict GPRs in radiotherapy dose verification for different anatomical structures, while providing interpretability analysis of model outcomes. By integrating patient CT images and RT dose, the method accurately predicts GPRs for PTV and OARs under various gamma criteria.

The GPR results reported in this study should be interpreted in the context of the specific dose calculation and gamma analysis settings. First, the dose calculation grid size was set to 2.5 mm, whereas gamma analysis was performed using distance‐to‐agreement (DTA) criteria of 2 and 3 mm. Thus, the spatial evaluation criteria were smaller than, or comparable to, the original dose sampling resolution. Although linear interpolation was applied during gamma calculation to partially mitigate this mismatch by estimating intermediate dose values, the reliability of the gamma results, particularly for the 2 mm DTA criterion, may still be reduced in regions with steep dose gradients and near small structures. Second, a global gamma method was used for gamma analysis, which may influence the gamma results in low‐dose regions, particularly in OARs. Therefore, the GPRs reported in this study, particularly under stringent gamma criteria, should be interpreted as relative indicators of agreement between dose distributions under identical dose calculation and gamma analysis settings, rather than as absolute measures of spatial dose accuracy. Finally, although AAPM TG‐218 recommends specific criteria for measurement‐based PSQA, the present study compared two calculated dose distributions. Therefore, the TG‐218 tolerance limits were not directly applied as clinical pass/fail thresholds, but were instead used to evaluate model performance under stringent validation conditions.

Radiomics employs automated algorithms to quantify phenotypic features in medical imaging, while dosimetrics focuses on quantifying phenotypic characteristics of radiation dose distribution.^34^, ^35^ Previous studies have demonstrated the predictive value of radiomic and dosimetric features in IMRT QA outcomes. Interian et al.^36^ employed convolutional neural networks to extract radiomic features from fluence maps, achieving an MAE as low as 0.70 under the 3%/3 mm criterion. Huang et al.^37^ utilized the UNet++ model to simultaneously process dose variations and gamma distribution maps, yielding an MAE of only 0.8% under the 3%/3 mm criterion. Although the above methods achieved good performance, truly three‐dimensional dose verification based on patient anatomy remains challenging in clinical practice. Most existing methods provide only limited planar dose information or rely on algorithms to reconstruct three‐dimensional dose distributions, making it difficult to comprehensively capture the complex heterogeneity of patient anatomy.^38^, ^39^ Therefore, in this study, we further integrated multi‐omics features based on the actual anatomical structures of different patients. The results showed that the multi‐omics model consistently outperformed single‐modality models. The fundamental reason is that radiomic and dosiomics features are clearly complementary in terms of the factors affecting the gamma passing rate: radiomic features reflect patient anatomy and tissue heterogeneity, whereas dosiomics features directly characterize the spatial pattern and complexity of the dose distribution. Joint modeling of these two types of features enables the simultaneous integration of biological information and dose‐spatial information, thereby providing a more comprehensive characterization of the key factors influencing the gamma passing rate. In future work, we will further introduce a measurement‐based PSQA system to conduct external validation in a representative subset of patients and compare the model predictions with actual measurement‐based verification results, so as to further evaluate the stability and reliability of the model in long‐term application.

This study confirms that ensemble learning models can effectively predict GPRs across different anatomical structures in IMRT plans. In comparison, the overall performance of the linear regression model was inferior to that of the ensemble learning models, with the relevant results provided in the Table S‐2. With increasing strictness of the gamma criteria, when the standard shifts from 3%/3 mm to 2%/2 mm, the prediction errors of all models generally increase, whereas the Spearman rank correlation coefficient decreases significantly. For example, for the PTV, under the 3%/3 mm standard, the RMSE on the test set was 2.95% ± 0.56%, and the MAE was 1.98% ± 0.31%. When the standard shifted to 2%/2 mm, the RMSE and MAE increased to 6.59% ± 0.84% and 4.97% ± 0.62%, respectively. This indicates that stricter gamma criteria amplify differences in dose distributions among radiotherapy plans, as well as uncertainties in treatment delivery, such as patient setup and organ motion, thereby increasing the difficulty of model prediction. Among different anatomical structures, the PTV exhibited the highest prediction errors across all regions, with the maximum values observed under the 2%/2 mm criterion (as detailed above). Prediction accuracy for OARs was generally higher than for the PTV. For example, under the 3%/3 mm criterion, the model achieved a minimum RMSE of 0.09% ± 0.02% and MAE of 0.06% ± 0.01% for the heart. This discrepancy likely stems from PTV regions typically exhibiting higher dose gradients, resulting in more nonlinear and challenging relationships between their radiomic features and GPRs. In contrast, dose distributions in OARs are relatively smoother and more stable, making their feature‐GPR correlations easier for models to learn. These phenomena align with the error increases observed under stringent standards by Huang et al.^37^and Lam et al.,^20^ while also corroborating Ishizaka et al.'s^22^finding that high‐complexity plans increase prediction uncertainty. The model constructed in this study has demonstrated good predictive performance; however, multicenter validation is still needed to further confirm its generalizability. Previous studies have explored this issue. For example, Valdes et al.^40^ developed a Poisson regression model combined with LASSO based on 498 IMRT plans from Institution 1 and externally validated it using 139 EPID measurements from Institution 2. The results showed that the model maintained relatively stable performance in cross‐center application, indicating a certain degree of generalizability. Therefore, multicenter validation will be an important step toward promoting the clinical application of the model developed in this study.

In this study, SHAP analysis was used to interpret the basis of model prediction at the feature level. Overall, texture heterogeneity‐related features played an important role across different organs and gamma criteria, suggesting that spatial complexity is an important source influencing GPR. This finding is consistent with previous studies showing that texture features can characterize the heterogeneity and complexity of dose distributions.^15^, ^41^, ^42^ Further analysis showed that, as the gamma criterion changed from 3%/3 mm to 2%/2 mm, the model's dependence on first‐order statistical features relatively decreased, whereas its dependence on higher‐order complex texture features increased. This suggests that local nonuniformity and subtle spatial differences become more critical for GPR prediction under stricter criteria. Although the key features differed to some extent among organs, they were generally concentrated on indicators reflecting gray‐level heterogeneity, texture complexity, and dose distribution morphology, indicating that GPR prediction is jointly influenced by organ‐specific anatomical background and local spatial characteristics.

Based on the feature selection results, PTV prediction was more strongly influenced by internal microstructural complexity, suggesting that tissue heterogeneity may further affect the results through dose calculation and irradiation conformity. Accordingly, in cases with low GPRs, further optimization of PTV contouring may be considered to: exclude non‐tumor heterogeneous tissues as much as possible, improve the accuracy of density assignment in heterogeneous regions, apply finer dose calculation resolution in areas with marked grayscale variations, and appropriately optimize MLC segmentation strategies to reduce potential calculation bias caused by complex target volumes. For lung tissue, the relevant features suggest that factors such as its low‐density background, coexistence of tumor and normal lung parenchyma, and uneven air cavity distribution may jointly increase dose calculation errors and aggravate the skewness of dose distribution. In such cases, beam angles and weights may be optimized clinically to reduce oblique beams passing through lung tissue. More refined lung contouring may also be considered to clearly distinguish tumor, normal lung parenchyma, and air cavities, with more accurate tissue density assignment applied during dose calculation. In addition, finer dose calculation grids or heterogeneity correction algorithms may be used for lung regions to reduce calculation bias caused by density differences. For cases obviously affected by respiratory motion, 4D‐CT or respiratory gating techniques may also be incorporated to reduce the impact of motion on dose distribution in lung tissue. For the heart, the relevant features indicate that dose distribution skewness, overall density characteristics, and local structural complexity may all contribute to GPR determination. This suggests that performance in the heart region is not only related to the mean dose level, but may also be closely associated with the smoothness of the local dose distribution and the presence of small‐scale abrupt changes. Accordingly, more refined heart contouring may be considered clinically to distinguish different structures, such as the cardiac chambers and myocardium, thereby reducing calculation errors caused by tissue density differences. Beam paths may also be optimized to reduce high‐angle beams passing through the heart, and dose constraints for cardiac substructures may be strengthened during plan design to avoid local overdosage or underdosage. For the spinal cord, the relevant features suggest that its performance may be more strongly influenced by the stability of the continuous spatial dose distribution. Because the spinal cord has a long and narrow anatomical shape, and clinical plans usually require a steep dose fall‐off around it, any decrease in dose distribution continuity may more readily lead to local dose deviations at the boundary or along the longitudinal axis. In view of this characteristic, more refined spinal cord contouring may be considered clinically to ensure clear boundaries and reduce partial‐volume effects. MLC shielding strategies may also be optimized to maintain dose uniformity along the longitudinal axis of the spinal cord as much as possible. When necessary, small‐field dose calibration may be incorporated to further improve dose calculation accuracy in the spinal cord region.

Based on this, the role of the predicted results in the actual clinical workflow should be further clarified. By using patient‐specific CT images and dose maps to predict GPRs, this study enables PSQA assessment based on patient anatomy. In clinical workflows, clinical decisions can generally be divided into three categories: (1) plan pass; (2) further measurement‐based verification required; and (3) plan failure. Future studies will explore the feasibility of converting predicted results into pass/fail classifications based on current thresholds, and further evaluate their practical applicability in clinical workflows. In addition, since threshold settings vary among centers, plan classification should be determined according to the GPR criteria used at each institution.

This study has several limitations. First, the model was developed using the currently available datasets, and potential data imbalances may restrict its predictive accuracy. Second, all data originated from a single institution and included only lung and esophageal cancer patients. Their anatomical structures, dose distributions, and extracted radiomics and dosimetric features differ from tumors in regions such as the head and neck or pelvis. Furthermore, the model's adaptability and robustness across different treatment center equipment configurations and multiple MLC models remain inadequately validated. Therefore, future research will utilize multi‐center collaboration and transfer learning strategies to validate and adapt the model to different prescriptions, anatomical sites, and devices, thereby enhancing its universality and clinical application value.

## 5 CONCLUSION

In this study, we developed and validated a predictive framework integrating multi‐omics features with interpretable machine learning to forecast GPRs in IMRT across various patient anatomical structures. The model that was created had a high predictive power. SHAP analysis showed that texture features were relatively important predictors of GPRs. Notably, the model automatically adjusted its dependence on the type of features depending on the anatomical situation‐ including dose gradients of the PTV and the low‐density properties of lung tissue‐ and different gamma criteria, which improved the explainability of the model predictions.

## AUTHOR CONTRIBUTIONS

**Xuerou Zhang**: analysis and interpretation of data. **Ying Huang and Xuerou Zhang**: drafted the manuscript. **Xingtong Zhang, Jie Wang and Yehui Luo**: revising the manuscript critically for important intellectual content. **Hua Chen and Jianhao Xie**: data/evidence collection. **Zhiyong Xu**: specifically critical review. **Yunhua Xu**: specifically critical review.

## CONFLICT OF INTEREST STATEMENT

The authors declare no conflicts of interest.

## ETHICS STATEMENT

This retrospective study was approved by the Ethics Committee of Shanghai Chest Hospital (approval number: KS24019). The ethics committee waived the requirement for informed consent due to the retrospective nature of the study and the use of anonymized patient data.

## Supporting information




**Supporting Information**: 2026‐09176‐sup‐0002‐S.docx

**Supporting Information**: 2026‐09176‐sup‐0003‐S.docx

**Supporting Information**: 2026‐09176‐sup‐0004‐S.docx

## Data Availability

The datasets generated during and/or analyzed during the current study are available from the corresponding author on reasonable request. The data are not publicly available due to participant privacy concerns. For further inquiries, please contact Yunhua Xu at 728001506@shsmu.edu.cn.
