# Supplementary material for: Explainable machine learning for patient‐specific quality assurance in intensity‐modulated radiotherapy based on anatomical structures
Source: J Appl Clin Med Phys. 2026 Jun 24;27(7):e70667. doi: 10.1002/acm2.70667 (PMC13292390; doi:10.1002/acm2.70667)
Supplement: Supplementary file 1 — Supporting Information: 2026‐09176‐sup‐0002‐S.docx [file ACM2-27-e70667-s002.docx]

**Supplementary Material**

Table S-1. Pyradiomics parameters

| Parameter category | Parameter | Setting |
| --- | --- | --- |
| Image type | Original | Enabled |
| Image filterin | LoG, Wavelet, LBP3D, etc. | Not used |
| Voxel resampling | Resampled pixel spacing | 1× 1×1 mm |
| Interpolation | Interpolator | sitkNearestNeighbor |
| Mask correction | correctMask | true |
| Bin width | binWidth | 5 |
| Feature classes | First-order, GLCM, GLRLM, GLSZM, GLDM, NGTDM | Enabled |

Table S-2. Prediction regression metrics of linear regression for PTV and OAR under different gamma threshold criteria.

| Organ | | MAE  (%) | RMSE  (%) |
| --- | --- | --- | --- |
| PTV | 3%/3 mm test | 7.26 ± 0.46 | 7.65 ± 0.36 |
|  | 3%/2 mm test | 6.48 ± 0.41 | 7.10 ± 0.38 |
|  | 2%/3 mm test | 6.22 ± 0.45 | 7.07 ± 0.60 |
|  | 2%/2 mm test | 5.81 ± 0.66 | 7.17 ± 0.88 |
| Lung_L | 3%/3 mm test | 3.74 ± 0.90 | 3.83 ± 0.85 |
|  | 3%/2 mm test | 5.93 ± 0.68 | 6.09 ± 0.65 |
|  | 2%/3 mm test | 5.89 ± 1.12 | 6.08 ± 1.01 |
|  | 2%/2 mm test | 8.13 ± 0.54 | 8.48 ± 0.47 |
| Lung_R | 3%/3 mm test | 2.10 ± 0.55 | 2.17 ± 0.52 |
|  | 3%/2 mm test | 4.27 ± 0.77 | 4.42 ± 0.72 |
|  | 2%/3 mm test | 3.28 ± 0.54 | 3.43 ± 0.52 |
|  | 2%/2 mm test | 5.99 ± 0.55 | 6.08 ± 0.11 |
| Total Lung | 3%/3 mm test | 1.38 ± 0.19 | 1.42 ± 0.18 |
|  | 3%/2 mm test | 2.07 ± 0.17 | 2.17 ± 0.15 |
|  | 2%/3 mm test | 1.89 ± 0.27 | 2.00 ± 0.24 |
|  | 2%/2 mm test | 2.47 ± 0.18 | 2.71 ± 0.14 |
| Heart | 3%/3 mm test | 0.35 ± 0.07 | 0.36 ± 0.06 |
|  | 3%/2 mm test | 0.35 ± 0.07 | 0.36 ± 0.06 |
|  | 2%/3 mm test | 0.51 ± 0.04 | 0.55 ± 0.04 |
|  | 2%/2 mm test | 4.94 ± 2.04 | 5.16 ± 1.71 |
| Spinal cord | 3%/3 mm test | 0.43 ± 0.04 | 0.45 ± 0.04 |
|  | 3%/2 mm test | 1.11 ± 0.09 | 1.14 ± 0.08 |
|  | 2%/3 mm test | 1.01 ± 0.03 | 1.05 ± 0.03 |
|  | 2%/2 mm test | 2.79 ± 0.27 | 2.88 ± 0.25 |

**Abbreviations:** PTV：planning target volume; Lung _L:Left Lung; Lung_R: right Lung；MAE, mean absolute error; RMSE, root mean squared error


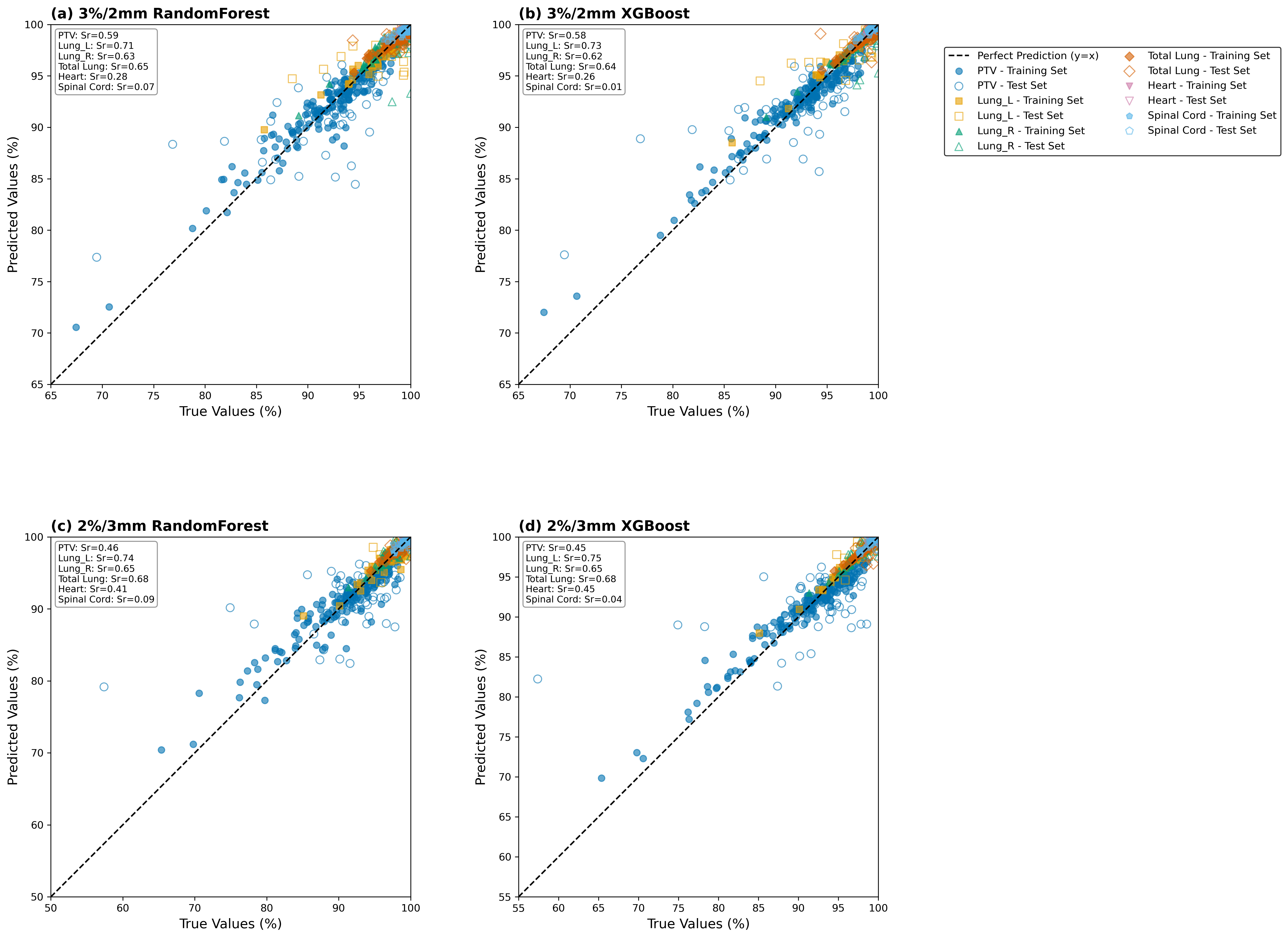


Figure S-1. Scatter distribution plots of the Random Forest and XGBoost models under the 3%/2 mm and 2%/3 mm gamma criteria: (a) 3%/2 mm Random Forest; (b) 3%/2 mm XGBoost; (c) 2%/3 mm Random Forest; (d) 2%/3 mm XGBoost.


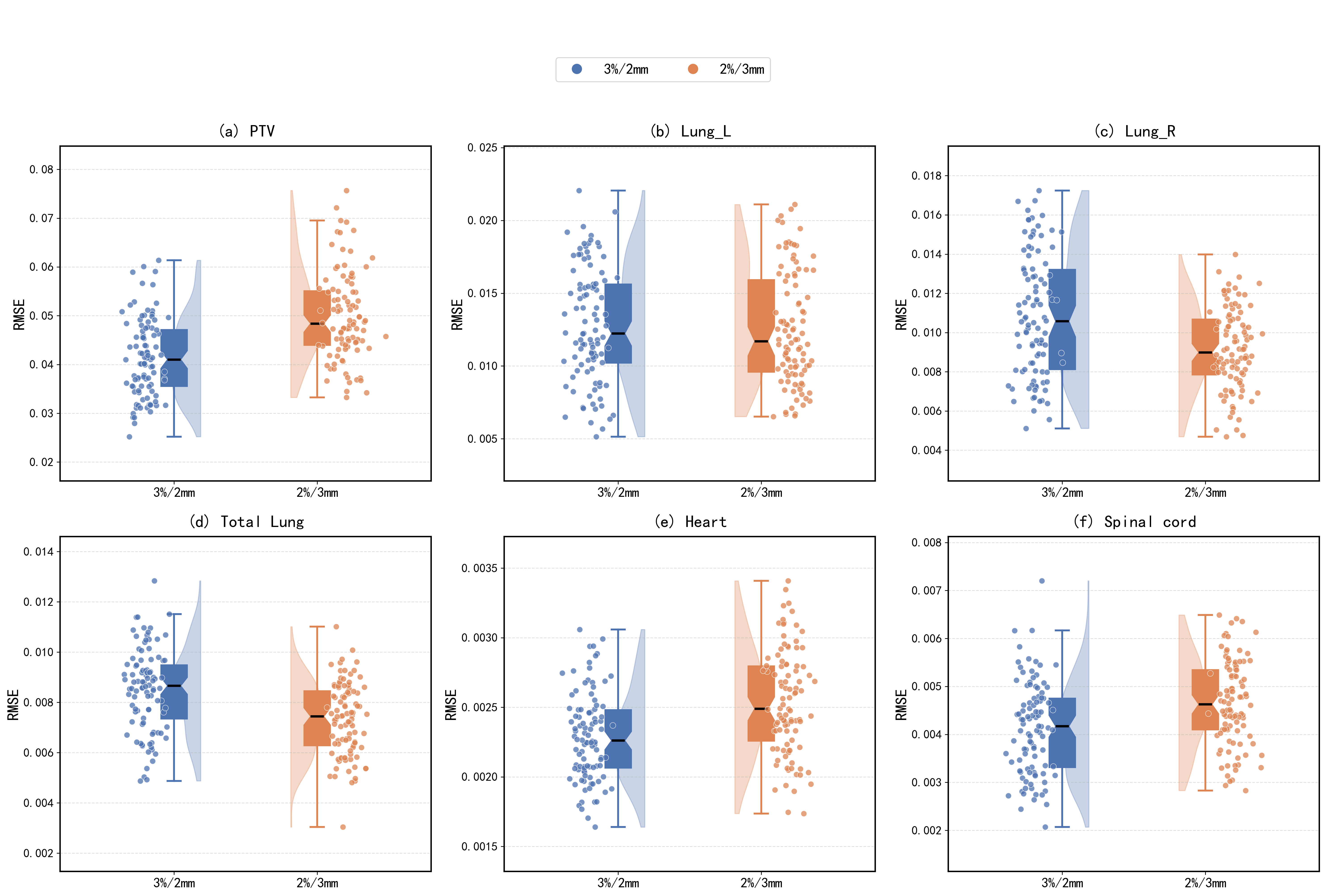


Figure S-2. Distribution plots of prediction errors for the PTV and organs at risk using the XGBoost model under the 3%/2 mm and 2%/3 mm gamma criteria: (a) PTV; (b) left lung; (c) right lung; (d) both lungs; (e) heart; (f) spinal cord.


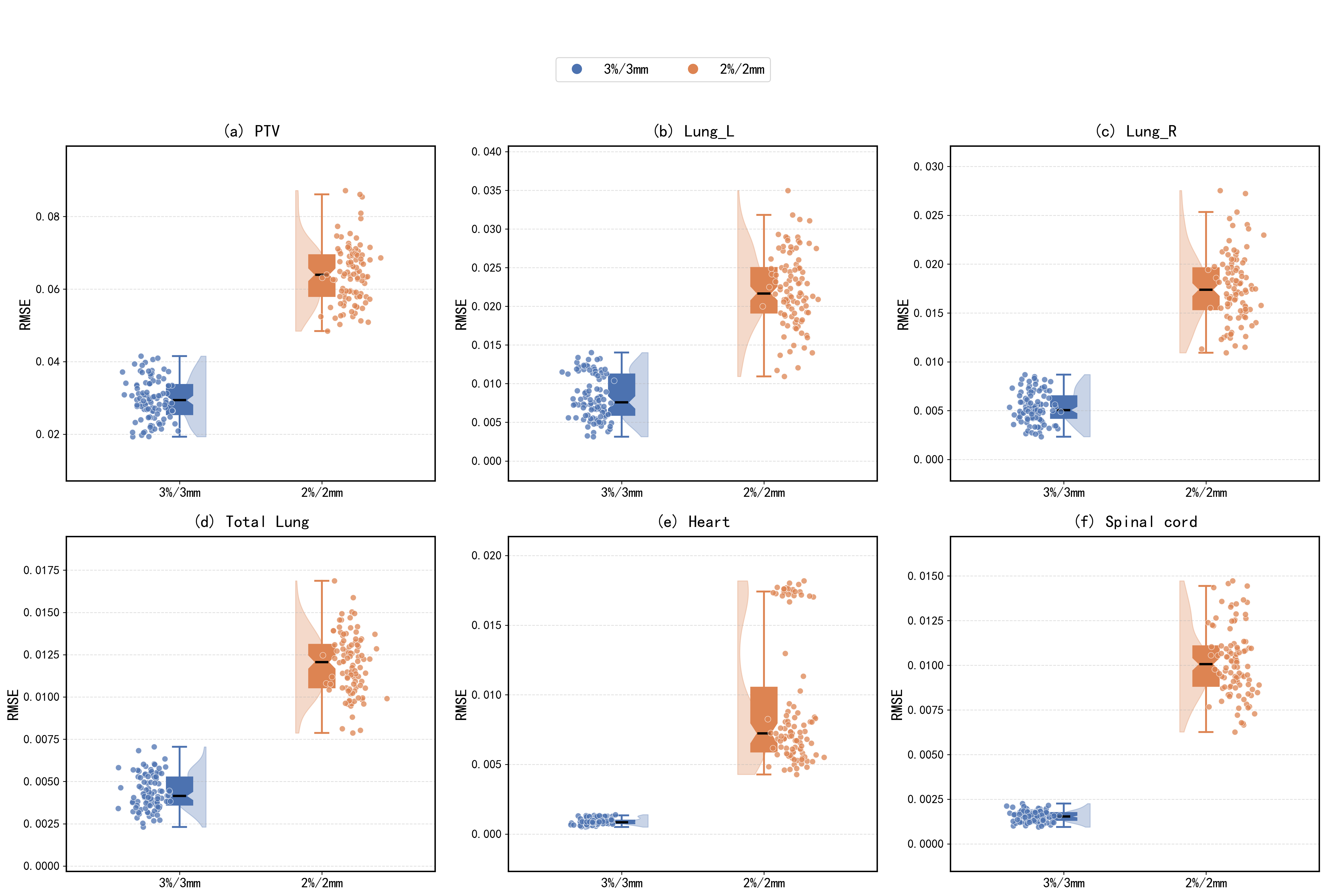


Figure S-3. Distribution plots of prediction errors for the PTV and organs at risk using the Random Forest model under the 3%/3 mm and 2%/2 mm gamma criteria: (a) PTV; (b) left lung; (c) right lung; (d) total lung; (e) heart; (f) spinal cord.


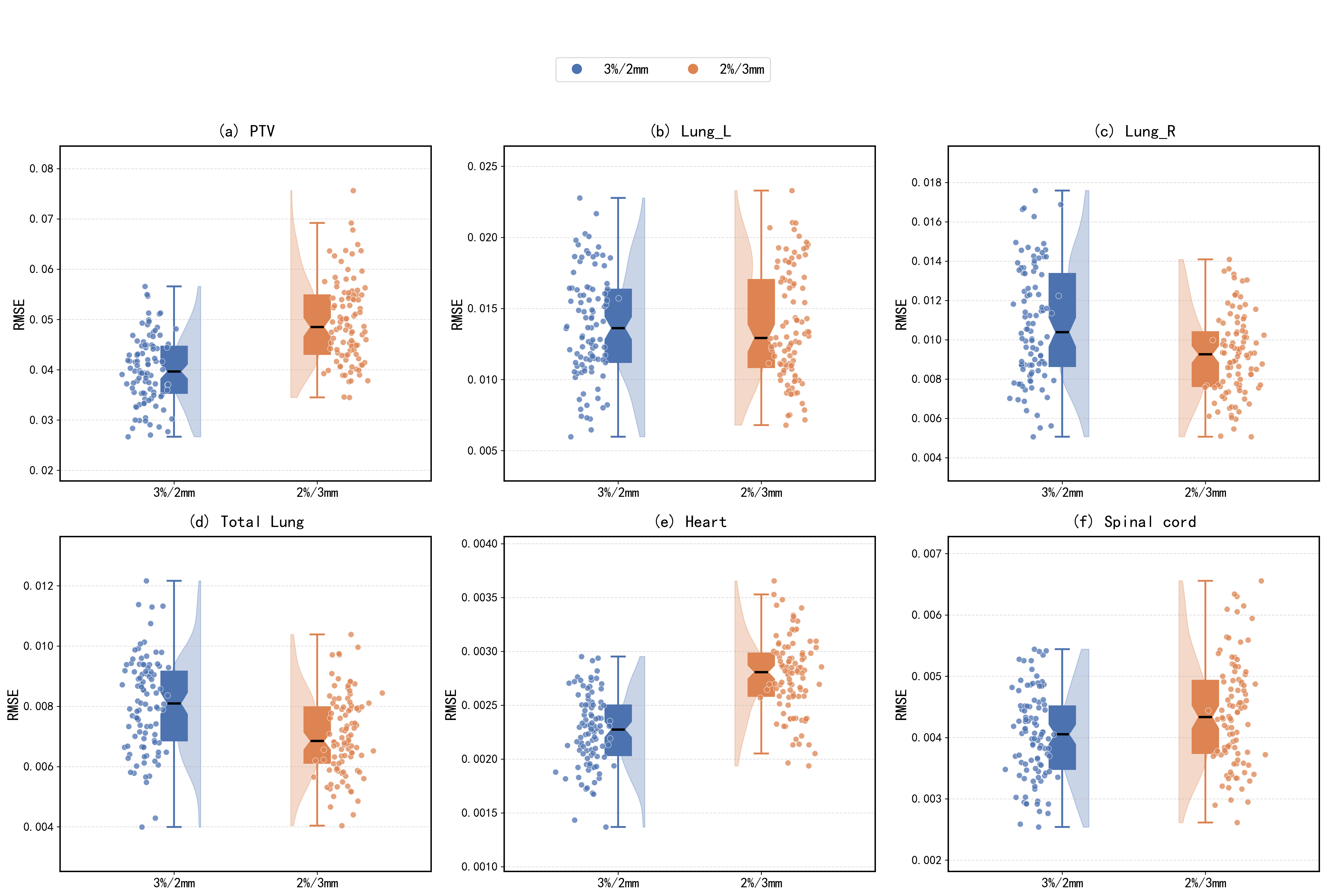


Figure S-4. Distribution plots of prediction errors for the PTV and organs at risk using the Random Forest model under the 3%/2 mm and 2%/3 mm gamma criteria: (a) PTV; (b) left lung; (c) right lung; (d) total lung; (e) heart; (f) spinal cord.
