# Supplementary material for: Explainable machine learning for patient‐specific quality assurance in intensity‐modulated radiotherapy based on anatomical structures
Source: J Appl Clin Med Phys. 2026 Jun 24;27(7):e70667. doi: 10.1002/acm2.70667 (PMC13292390; doi:10.1002/acm2.70667)
Supplement: Supplementary file 2 — Supporting Information: 2026‐09176‐sup‐0003‐S.docx [file ACM2-27-e70667-s003.docx]

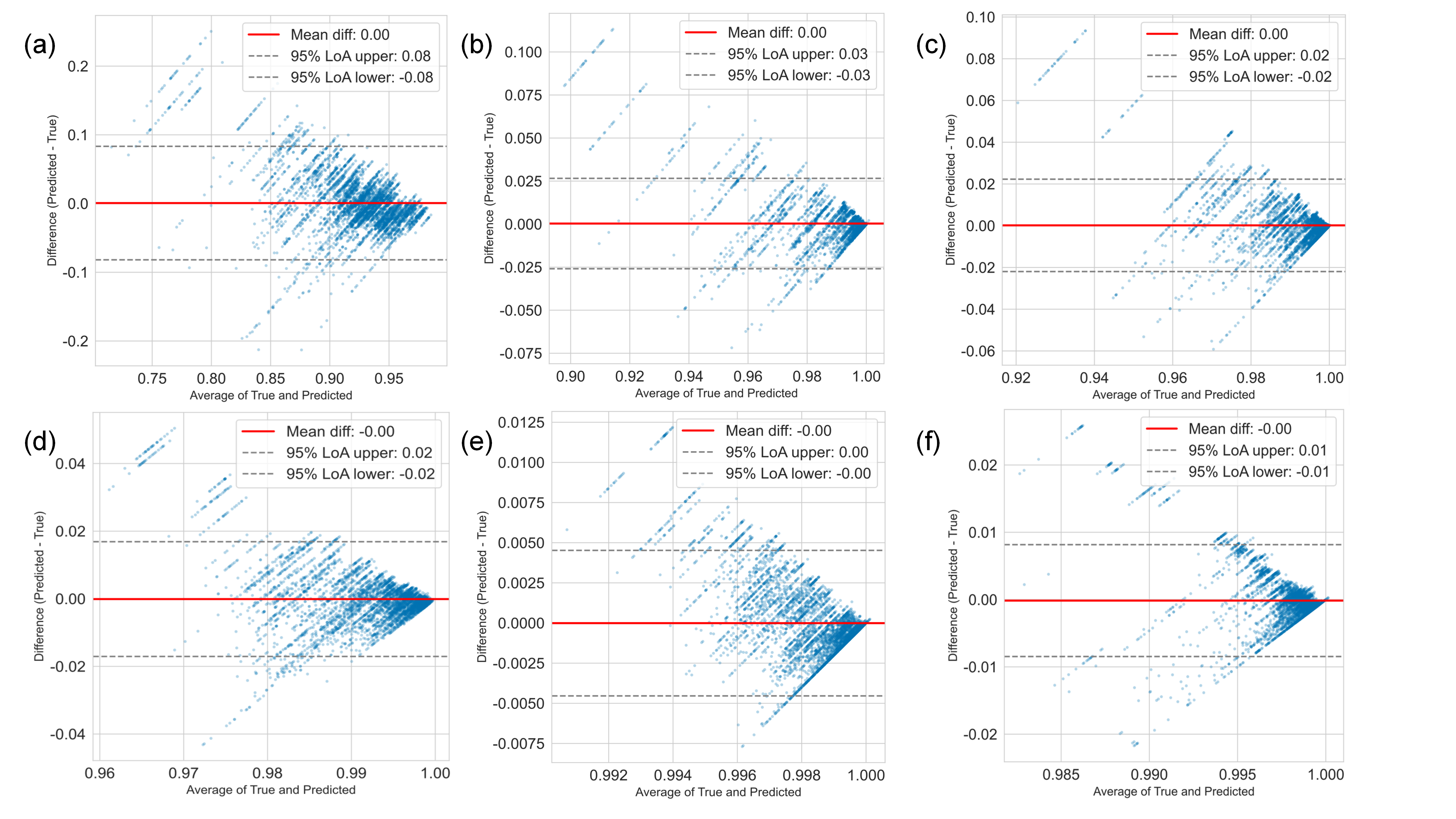


Figure S-5. Bland–Altman analysis of XGBoost under the 3%/2 mm gamma criterion: (a) PTV; (b) left lung; (c) right lung; (d) total lung; (e) heart; (f) spinal cord.


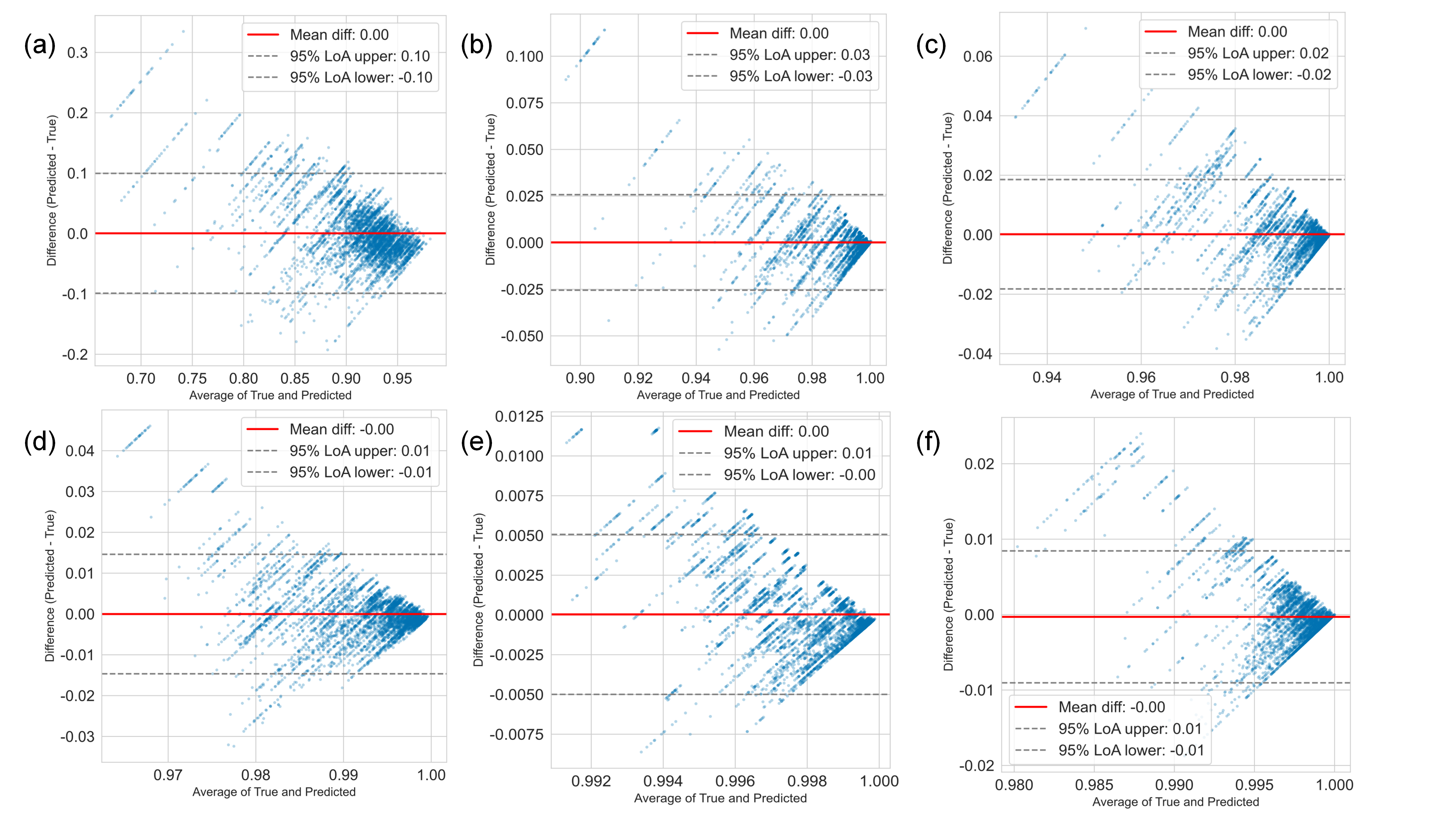


Figure S-6. Bland–Altman analysis of XGBoost under the 2%/3 mm gamma criterion: (a) PTV; (b) left lung; (c) right lung; (d) total lung; (e) heart; (f) spinal cord.


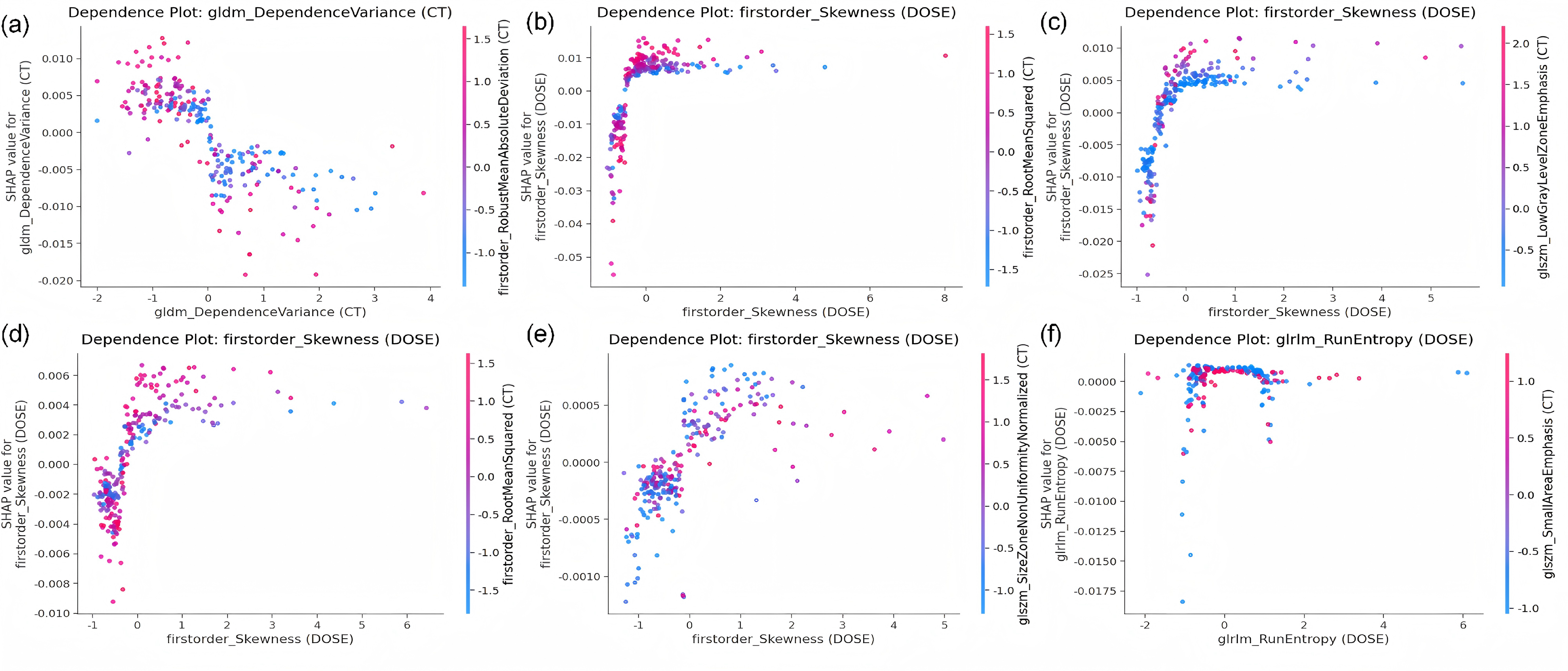


Figure S-7. SHAP dependence plots of the main features for XGBoost under the 3%/2 mm gamma criterion: (a) PTV; (b) left lung; (c) right lung; (d) total lung; (e) heart; (f) spinal cord.


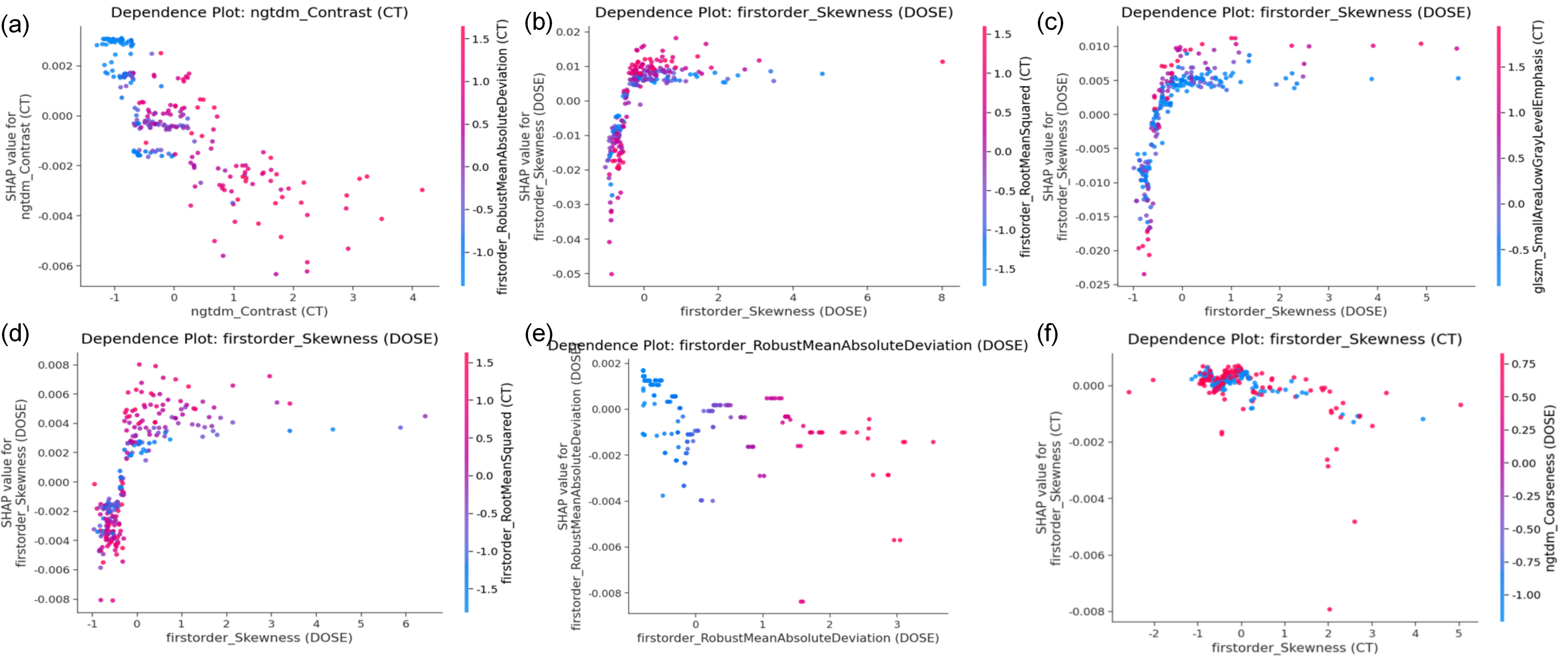


Figure S-8. SHAP dependence plots of the main features for XGBoost under the 2%/3 mm gamma criterion: (a) PTV; (b) left lung; (c) right lung; (d) total lung; (e) heart; (f) spinal cord.


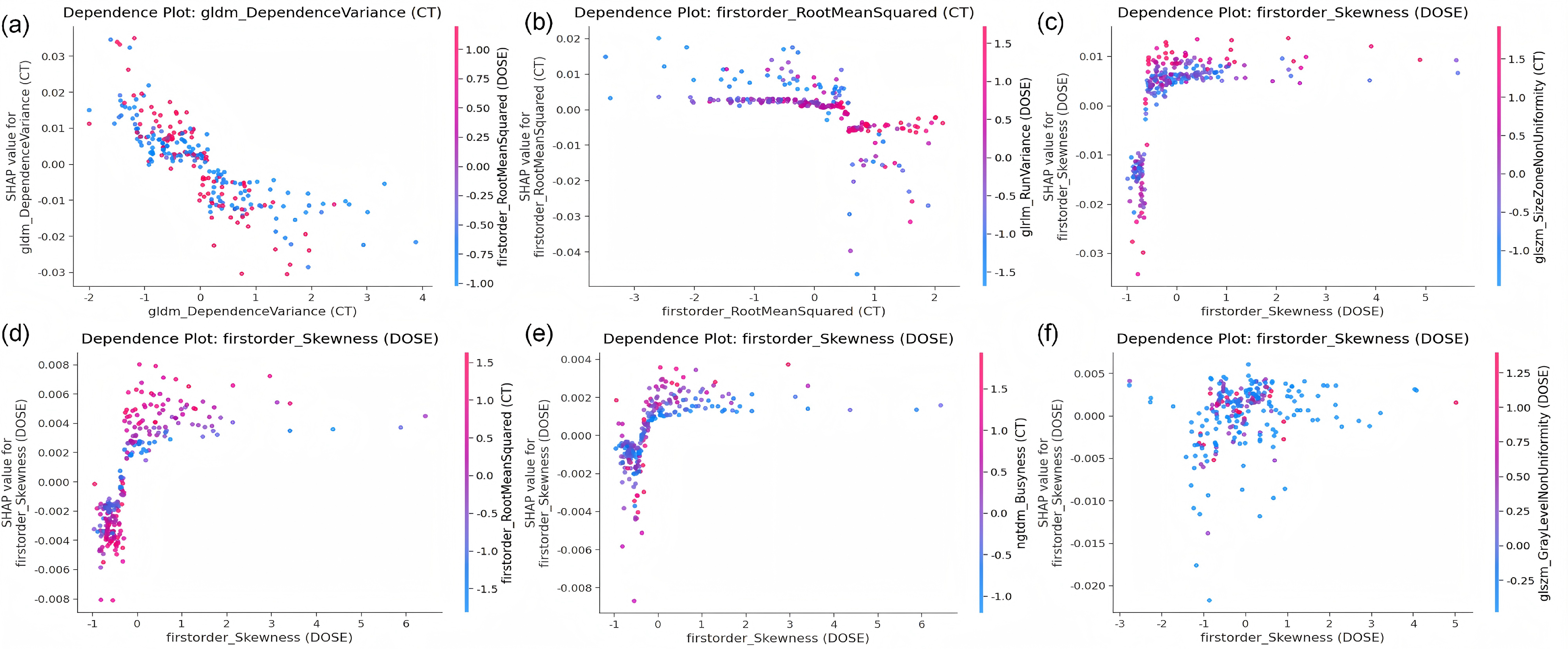


Figure S-9. SHAP dependence plots of the main features for XGBoost under the 2%/2 mm gamma criterion: (a) PTV; (b) left lung; (c) right lung; (d) total lung; (e) heart; (f) spinal cord.
