# Supplementary material for: Explainable machine learning for patient‐specific quality assurance in intensity‐modulated radiotherapy based on anatomical structures
Source: J Appl Clin Med Phys. 2026 Jun 24;27(7):e70667. doi: 10.1002/acm2.70667 (PMC13292390; doi:10.1002/acm2.70667)
Supplement: Supplementary file 3 — Supporting Information: 2026‐09176‐sup‐0004‐S.docx [file ACM2-27-e70667-s001.docx]

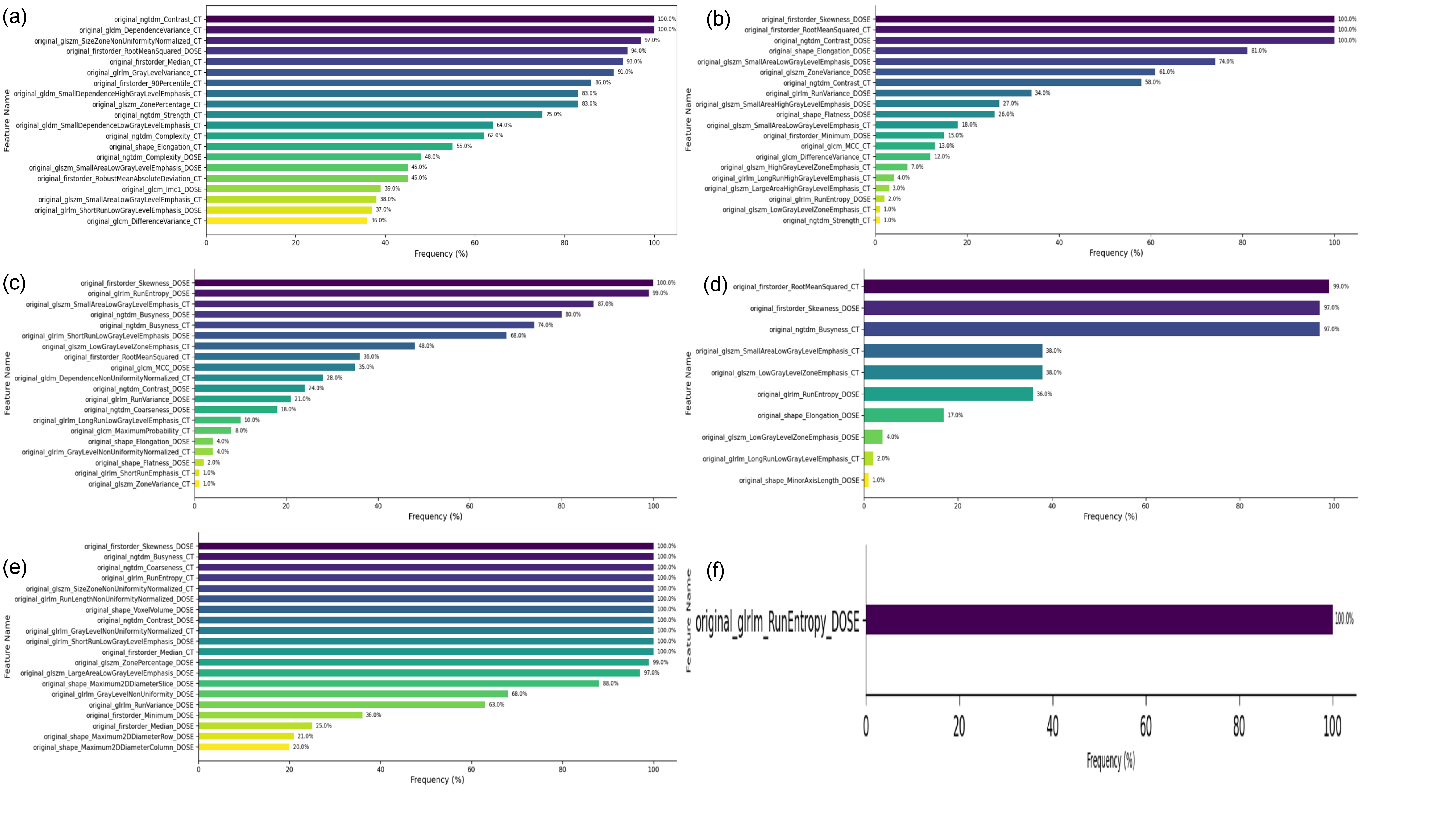


Figure S-10. Selection frequency of the main features for XGBoost under the 3%/3 mm gamma criterion: (a) PTV; (b) left lung; (c) right lung; (d) total lung; (e) heart; (f) spinal cord.


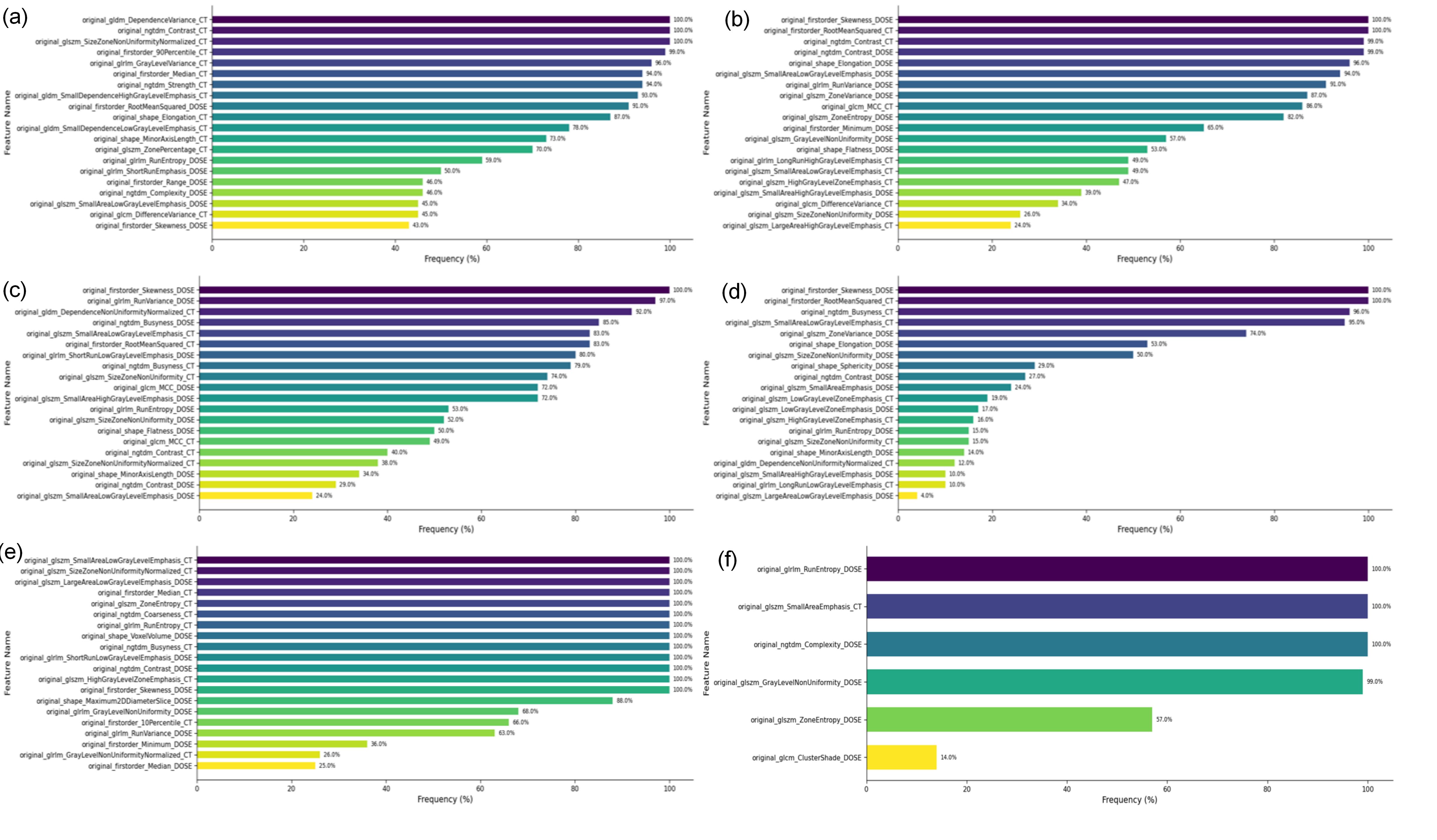


Figure S-11. Selection frequency of the main features for XGBoost under the 3%/2 mm gamma criterion: (a) PTV; (b) left lung; (c) right lung; (d) total lung; (e) heart; (f) spinal cord.


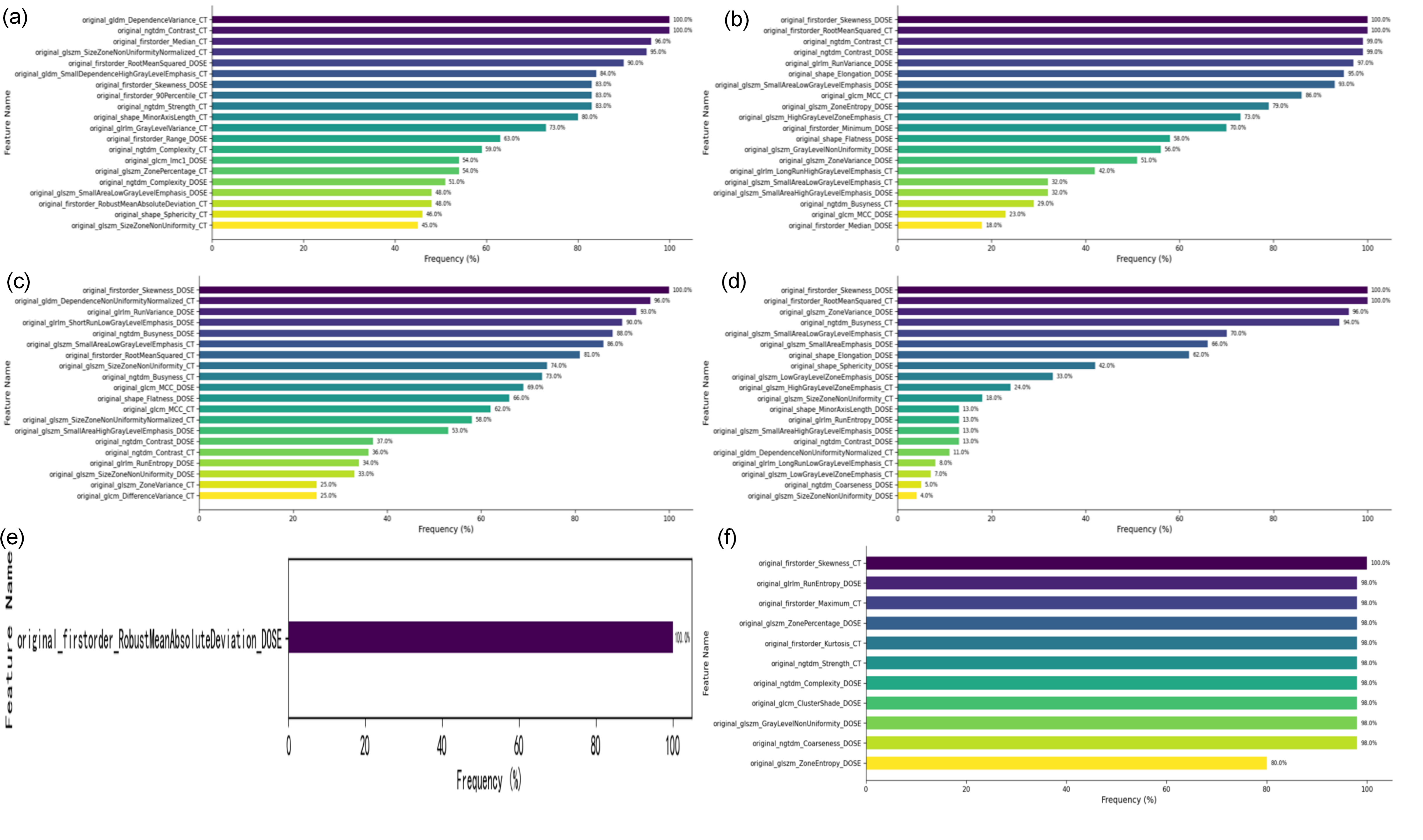


Figure S-12. Selection frequency of the main features for XGBoost under the 2%/3 mm gamma criterion: (a) PTV; (b) left lung; (c) right lung; (d) total lung; (e) heart; (f) spinal cord.


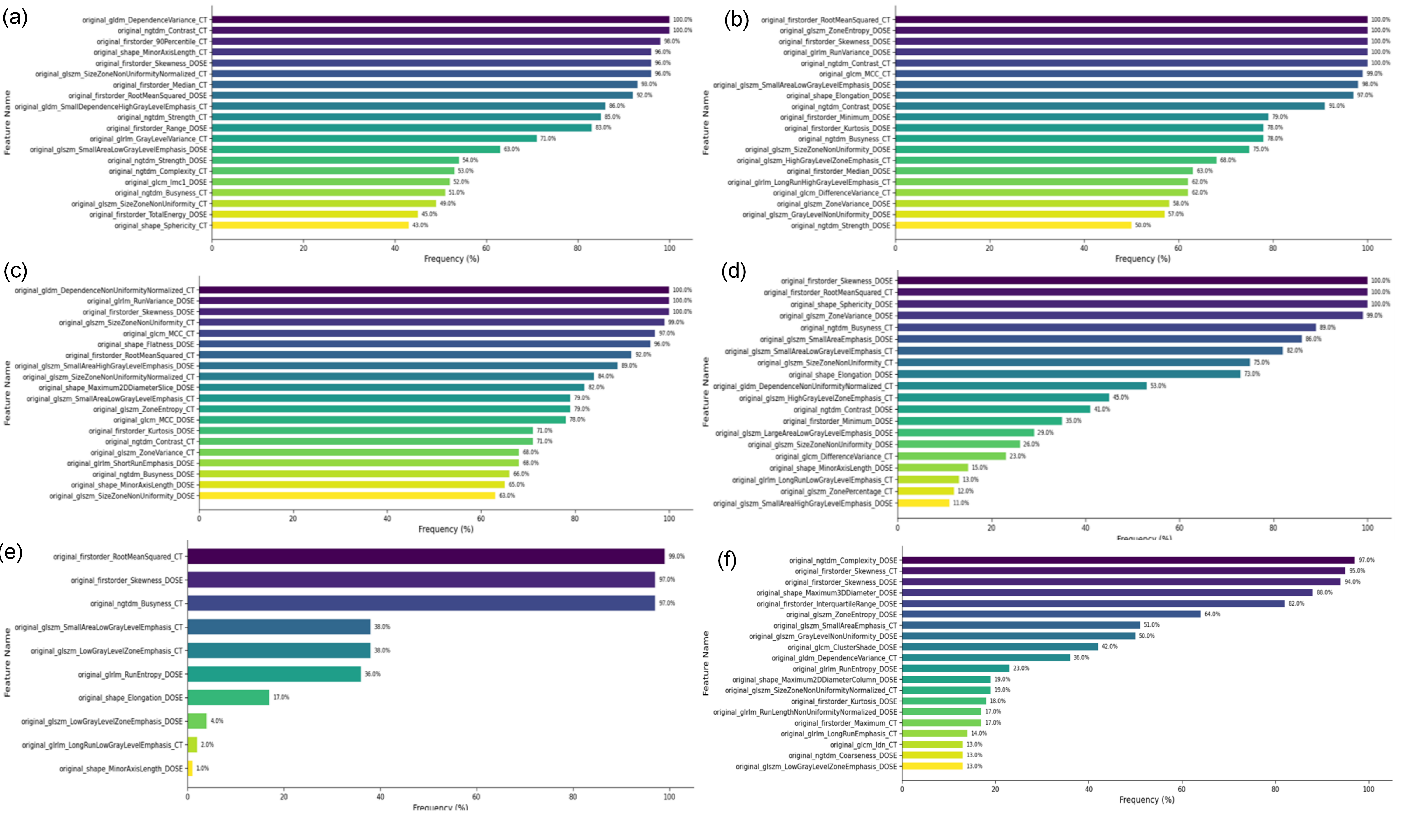


Figure S-13. Selection frequency of the main features for XGBoost under the 2%/2 mm gamma criterion: (a) PTV; (b) left lung; (c) right lung; (d) total lung; (e) heart; (f) spinal cord.
